# Integrated machine learning methods identify FNDC3B as a potential prognostic biomarker and correlated with immune infiltrates in glioma

**DOI:** 10.3389/fimmu.2022.1027154

**Published:** 2022-10-06

**Authors:** Xiao Wang, Yeping Huang, Shanshan Li, Hong Zhang

**Affiliations:** ^1^ Department of Nephrology, Sir Run Run Shaw Hospital, Zhejiang University School of Medicine, Hangzhou, China; ^2^ Shanghai Diabetes Institute, Shanghai Jiao Tong University Affiliated Sixth People’s Hospital, Shanghai, China

**Keywords:** FNDC3B, glioma, prognosis, immune infiltration, The Cancer Genome Atlas (TCGA), Chinese Glioma Genome Atlas (CGGA)

## Abstract

**Background:**

Recent discoveries have revealed that fibronectin type III domain containing 3B (FNDC3B) acts as an oncogene in various cancers; however, its role in glioma remains unclear.

**Methods:**

In this study, we comprehensively investigated the expression, prognostic value, and immune significance of FNDC3B in glioma using several databases and a variety of machine learning algorithms. RNA expression data and clinical information of 529 patients from the Cancer Genome Atlas (TCGA) and 1319 patients from Chinese Glioma Genome Atlas (CGGA) databases were downloaded for further investigation. To evaluate whether FNDC3B expression can predict clinical prognosis of glioma, we constructed a clinical nomogram to estimate long-term survival probabilities. The predicted nomogram was validated by CGGA cohorts. Differentially expressed genes (DEGs) were detected by the Wilcoxon test based on the TCGA-LGG dataset and the weighted gene co-expression network analysis (WGCNA) was implemented to identify the significant module associated with the expression level of FNDC3B. Furthermore, we investigated the correlation between FNDC3B with cancer immune infiltrates using TISIDB, ESTIMATE, and CIBERSORTx.

**Results:**

Higher FNDC3B expression displayed a remarkably worse overall survival and the expression level of FNDC3B was an independent prognostic indicator for patients with glioma. Based on TCGA LGG dataset, a co-expression network was established and the hub genes were identified. FNDC3B expression was positively correlated to the tumor-infiltrating lymphocytes and immune infiltration score, and high FNDC3B expression was accompanied by the increased expression of B7-H3, PD-L1, TIM-3, PD-1, and CTLA-4. Moreover, expression of FNDC3B was significantly associated with infiltrating levels of several types of immune cells and most of their gene markers in glioma.

**Conclusion:**

This study demonstrated that FNDC3B may be involved in the occurrence and development of glioma and can be regarded as a promising prognostic and immunotherapeutic biomarker for the treatment of glioma.

## Introduction

Glioma is the most common primary tumor of the central nervous system in adults and is characterized by high recurrence and mortality rates. According to the World Health Organization (WHO), glioma is typically divided into two principal subgroups: low-grade glioma (LGG; grade II and III) and glioblastoma multiforme (GBM; grade IV; most aggressive and lethal subtype) based on the malignant degree (1). The median survival is less than two years, and the overall prognosis is poor for glioma patients even after surgical resection, chemotherapy, and radiation therapy (CRT), especially for those with GBM (2). Even though many advances have been made in adjuvant therapy and surgery in the past few decades, the clinical outcomes have not been significantly improved for glioma patients. Apart from the traditional treatment (3), studies in recent years have revealed the use of novel and effective methods, such as immunotherapy to treat glioma owing to the success achieved from other solid tumors, including lung, bladder, and kidney cancers, and melanoma (4, 5). However, there is still an urgent need to identify additional immune biomarkers for combination therapy due to the resistance to monotherapy (6–8). Furthermore, this may elucidate the mechanism of tumorigenesis and help to identify new molecular targets for treatment.

Fibronectin type III domain containing 3B (FNDC3B, also named FAD104), which belongs to the FNDC3 family, was initially identified as a regulator of adipocyte and osteoblast differentiation (9). FNDC3B is an endoplasmic reticulum transmembrane protein with a single transmembrane domain at the C terminus preceded by nine repeated fibronectin type III domains. Its biological function remains largely unknown (10). Recent research demonstrated that FNDC3B plays a major role in cell adhesion, proliferation, and growth signaling due to the fibronectin type III domain, which has the ability to combine with various proteins (11). For the past few years, emerging evidence has demonstrated that FNDC3B was abnormally expressed in several types of human cancers, including hepatocellular carcinoma, acute myeloid leukemia, colorectal and cervical cancers (12–15). For instance, Han reported that FNDC3B expression was correlated with a worse prognosis in cervical cancer, while its carcinogenic effects are still unclear (15). Notably, a few studies have demonstrated that FNDC3B expression levels are correlated with glioblastoma. Wang and Xu reported that MiR-1225-5p and MiR-129-5p inhibit the malignant glioblastoma cells *via* targeting FNDC3B (16, 17). Furthermore, a newly integrated analysis of RNA binding proteins in glioma revealed that FNDC3B can not only serve as a useful prognostic biomarker but also promote glioma cell proliferation (18). However, the overall expression profile of FNDC3B and its potential role in the development and distinct clinical significance of glioma has not been fully elucidated. In previous study, Rajasagi et al. found that long-lived cytotoxic T-cell responses against peptides generated from personal tumor mutations in FNDC3B presented on chronic lymphocytic leukemia cells (19). Until now, there are very limited studies on the correlation between FNDC3B and tumor-infiltrating lymphocytes (TILs) in glioma.

Recent advancements in high-throughput sequencing technologies and large-scale cancer genomics databases have enabled a systematic and comprehensive analysis of genes from the perspective of machine learning (20–22). In the present study, we carried out an intensive analysis for the expression signature of FNDC3B using various publicly accessible databases, as well as applied data mining of the TCGA and CGGA datasets to explore its in-depth prognostic effect. We also investigated the correlations between FNDC3B expression and tumor immune microenvironment (TIM) in LGG patients in order to elucidate the underlying mechanisms and improve molecular diagnosis for glioma patients.

## Materials and methods

### Gene expression pattern based on ONCOMINE and GEPIA2

FNDC3B expression levels in various cancers were firstly explored by ONCOMINE database (https://www.oncomine.org/) (23), which is currently the largest public cancer microarray database and integrated data-mining platform. The threshold for ONCOMINE was set according to the default settings of P < 0.0001, fold change > 2, and Gene Rank < Top 10%. Gene expression profiling interactive analysis 2 (GEPIA2) is another useful web-based tool (http://gepia.cancer-pku.cn/) that contains RNA sequencing data based on 9,736 tumor and 8,587 normal control samples from The Cancer Genome Atlas (TCGA) and The Genotype-Tissue Expression (GTEx) (24), providing differential expression analysis, correlation analysis, survival analysis, and custom data analysis. GEPIA2 contains 518 LGG samples, 163 GBM samples, and 207 normal brain samples. FNDC3B expression was compared between LGG or GBM and normal tissues by Student t-tests. Samples were considered to be significant with p < 0.05 and fold change > 2. Protein expression of FNDC3B in glioma and normal brain tissues were evaluated based on immunohistochemistry data from the Human Protein Atlas (HPA) (https://www.proteinatlas.org/). The mRNA expression of FNDC3B in various human cancer cell lines were obtained from Broad Institute Cancer Cell Line Encyclopedia (CCLE).

### Data source and processing

**Figure 1:**
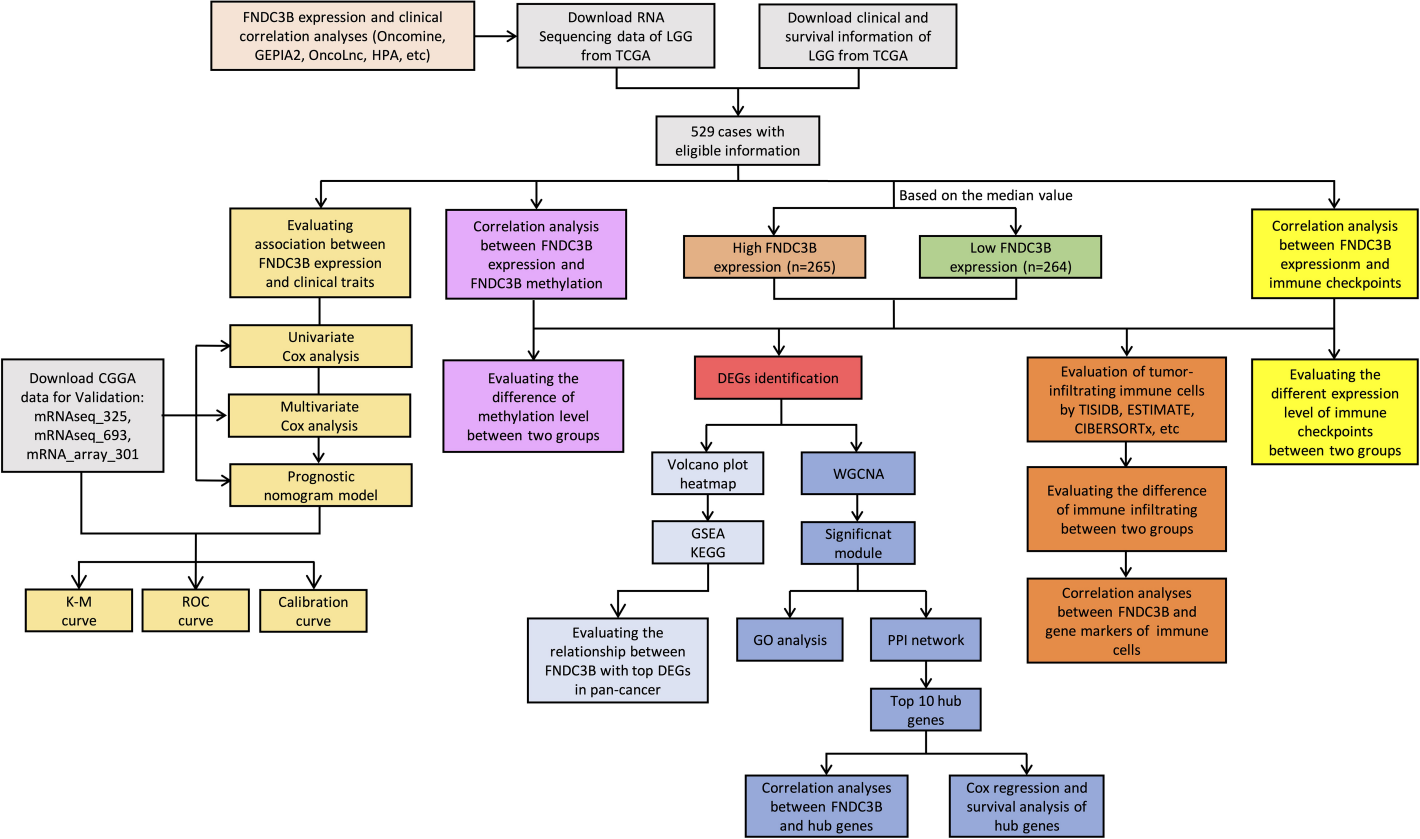
Flowchart of this study: data collection, processing, analysis, and validation. LGG, low-grade glioma; TCGA, The Cancer Genome Atlas; CGGA, Chinese Glioma Genome Atlas; GEPIA2, gene expression profiling interactive analysis 2; HPA, Human Protein Atlas; K-M, Kaplan-Meier; ROC, receiver operating characteristic; DEGs, differentially expressed genes; WGCNA, weighted gene co-expression network analysis; GSEA, gene set enrichment analysis; KEGG, kyoto encyclopedia of genes and genomes; GO, gene ontology; PPI, protein-protein interaction.

Gene expression data and corresponding clinical information for glioma patients were downloaded from TCGA (LGG and GBM) and Chinese Glioma Genome Atlas (CGGA) (mRNAseq_325, mRNAseq_693 and mRNA_array_301) database. The data from the TCGA was applied to explore the prognostic role of FNDC3B in gliomas, and the three CGGA cohorts were used to validate the results. The DNA methylation along with transcriptional data of FNDC3B for patients with LGG from the TCGA database was downloaded *via* the cBio Cancer Genomics Portal (cBioPortal) website (http://www.cbioportal.org/). The detailed methodology of the study is shown in **Figure 1**.

### Clinicopathological correlation and prognosis analysis

Coefficients of Cox regression were determined by data mining among 21 TCGA cancer types using OncoLnc (http://www.oncolnc.org/) to compare FNDC3B expression in different tumors. The gene expression data and corresponding clinical information from the TCGA and CGGA were used to evaluate the prognostic role of FNDC3B in gliomas. Patients with incomplete clinical information were eliminated. We explored the correlation between FNDC3B expression and various clinical features using the Wilcoxon test in R version 3.6.3. Kaplan-Meier analysis and log-rank test were implemented to evaluate the associations of FNDC3B and clinical variables with overall survival (OS). Univariate and multivariate Cox regression analyses were performed to further examine whether FNDC3B expression was a significant factor associated with OS when adjusted by clinical variables (age at initial pathological diagnosis, gender, neoplasm histologic grade, IDH mutation status, etc.). “Survival” and “survminer” packages in R software were used for the stepwise variable selection and Cox model construction.

### Construction and evaluation of a nomogram

Nomogram is widely used as a predictive model for cancer patients and can provide prognostic risk individually and intuitively (25). Based on the TCGA-LGG dataset, we constructed a prognostic nomogram model to predict the probability of 2-, 3-, and 5- year OS using “rms” package in R. The nomogram combined the expression level of FNDC3B with traditional clinical parameters (age, grade, and IDH status) and formulated the scoring criteria for all the parameters in the regression equation based on their regression coefficients. Then, the summed score for each patient was converted into the probability of the outcome time by using the nomogram. The performance and prediction efficiency of the nomogram were evaluated *via* plotting the calibration, K-M and receiver operating characteristic (ROC) curves of the three CGGA validation datasets.

### Screening of differentially expressed genes (DEGs)

The median value was used to create a categorical dependent variable based on FNDC3B expression level. For the TCGA-LGG dataset, DEGs between the high and low FNDC3B groups were screened using Wilcoxon test (screening criteria: P < 0.01, FDR < 0.05, and |logFC| > 1). Volcano plot of all DEGs was generated using R. For accurate results, we set the average expression level to at least 1 for the raw DEGs. Then, the filtered DEGs were selected for further analysis. Heatmap was generated by “ComplexHeatmap” package in R. To identify biological pathway differences between the high and low FNDC3B groups, gene set enrichment analysis (GSEA) and kyoto encyclopedia of genes and genomes (KEGG) were performed on the screened DEGs using “org.Hs.eg.db”, “clusterProfiler”, “enrichplot” and “ggplot2” R packages. The correlations between FNDC3B with DEGs in pan-cancer were obtained by using Gene_Corr module of TIMER2.0 (tumor immune estimation resource, version 2) (http://timer.cistrome.org/). P < 0.05 was considered to be a significant enrichment.

### Co-expression network creation and hub genes identification

To reveal the correlation between genes and identify the ones with significant relationships, 2,099 DEGs between the high and low FNDC3B expression groups were used to construct a weighted co-expression network. Patients with incomplete expression information were removed, and the remaining samples were implemented to construct the network. In order to ensure the reliability of the network structure, we set gene expression value larger than 1 in at least 10% of all the samples, and the average expression level was at least 0.5 for the raw DEGs. A scale-free gene co-expression network based on 673 filtered DEGs was constructed using the R package “WGCNA”. Hierarchical clustering tree was created based on a dissimilarity measure (1-TOM), and genes with similar expression patterns were merged into the same module. The most relevant module was revealed by calculating the correlation between modules and the FNDC3B group; the genes in the most significant module were extracted to determine the target genes. For the selected module, Gene Ontology (GO) was performed in R using the packages “org.Hs.eg.db”, “AnnotationDbi”, “enrichplot” and “ggplot2” with q-values less than 0.0001. The Search Tool for the Retrieval of Interacting Genes Database (STRING) version 11 was applied to generate the PPI network and the combined score > 0.4 was used as the cut-off criterion. Cytoscape version 3.8.2 was employed to visualize the molecular interaction networks and biological pathways. Hub genes were identified *via* CytoHubba plugin with the top 10 MCC values. We used GeneMANIA database (http://genemania.org/) to build the gene-gene interaction network for hub genes in terms of physical interactions, co-expression, shared protein domains, pathways, predicted interactions, and colocalization, as well as to predict their biological functions. The relationships of the expression levels between the hub genes and FNDC3B were shown by scatterplots using “ggplot2” R package.

### Analysis of FNDC3B-associated immunomodulators

To investigate the immune infiltration of FNDC3B in different cancers, TISIDB database (http://cis.hku.hk/TISIDB/) was applied to infer the correlations between 28 types of tumor-infiltrating lymphocytes (TILs) and FNDC3B expression. TISIDB integrates a variety of data sources in tumor immunology, including abundant human cancer datasets from TCGA and text mining results from PubMed. Spearman correlation test was implemented to estimate the association between FNDC3B and TILs. Furthermore, Estimation of Stromal and Immune cells in Malignant Tumor tissues using Expression (ESTIMATE) algorithm was employed to evaluate the stromal score, immune score, and ESTIMATE score for each sample using the downloaded data. Moreover, CIBERSORTx (https://cibersortx.stanford.edu/) was used to assess the relative variations of 22 types of tumor-infiltrating immune cells between the high and low FNDC3B expression groups in LGG. Packages “ggplot2”, “ggpubr” and “ggExtra” in R were applied to investigate the correlation between FNDC3B and immune checkpoints based on TCGA-LGG dataset. The associations between gene markers of the significant immune cells and FNDC3B expression were also determined by the correlation analysis function in GEPIA2. Differences with a P-value < 0.05 were considered significant in all tests.

### Statistical analysis

All statistical analysis was performed in R software and P < 0.05 was considered statistically significant. Wilcoxon test was used to compare the differences for clinical characteristics or immune scores grouped by FNDC3B expression. Univariate (Log-rank test) and multivariate Cox regression (cox proportional hazard, coxph) analyses were performed to assess clinical traits associated with OS.

## Results

### FNDC3B mRNA expression in various cancers

**Figure 2:**
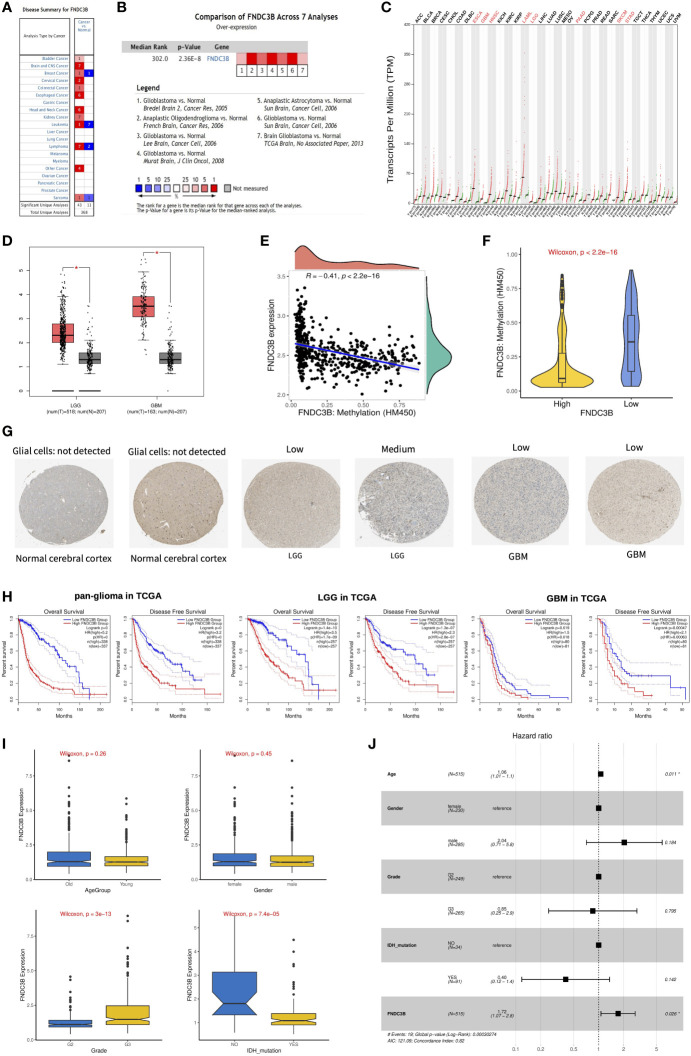
The expression profiles and prognostic value of FNDC3B in glioma. **(A)** In brain and CNS cancers, FNDC3B was significantly upregulated in seven studies. Red represents high expression and blue represents low expression. The darker the red color, the higher the gene expression level. The darker the blue color, the lower the gene expression level. **(B)** Comparison of FNDC3B expression across seven analyses, and red represents high expression. **(C)** FNDC3B was significantly upregulated in various tumors. **(D)** Expression level of FNDC3B in LGG and GBM compared to control. *, P < 0.05. **(E)** The expression of FNDC3B was negatively regulated by FNDC3B DNA methylation. **(F)** Different methylation levels of FNDC3B in the FNDC3B high- and low-expression groups in TCGA LGG samples. **(G)** Representative FNDC3B protein expression in normal and glioma tissues. Data were obtained from the Human Protein Atlas (HPA). **(H)** Kaplan-Meier analysis of OS and DFS based on FNDC3B high- vs. low-expression in pan-glioma, LGG, and GBM patients in the TCGA dataset. Red curve represents patients with high expression of FNDC3B, and blue curve represents low FNDC3B. **(I)** The correlations between FNDC3B expression and clinical characteristics based on TCGA LGG datasets: age, gender, grade, and isocitrate dehydrogenase (IDH) status. **(J)** Multiple Cox regression analysis of clinicopathological features (including FNDC3B expression) and OS in the TCGA LGG datasets.

We first assessed the expression of FNDC3B in different tumors and normal tissues of multiple cancer types using the Oncomine database. The results showed that abnormal FNDC3B expression was retrieved from a total of 368 datasets. Among them, FNDC3B expression levels were significantly upregulated in tumor tissues in 43 datasets, including brain and central nervous system (CNS), head and neck, esophageal, kidney, cervical, bladder, and colorectal cancers, etc. (**Figure 2A**, P < 0.0001, Fold Change > 2, and Gene Rank < Top 10%). In addition, its expression in leukemia, lymphoma, breast cancer, and sarcoma lymphoma was shown to be downregulated in multiple datasets. In summary, FNDC3B is generally upregulated in several tumors. In the brain and CNS cancers dataset, there were seven studies on the upregulation of FNDC3B and no studies on its downregulation. Comparison of FNDC3B across the seven studies showed a Median Rank = 302, suggesting that FNDC3B was highly expressed in glioma tissues and concentrated both in LGG and GBM (**Figure 2B**, P <0.0001). As for the outlier analysis of FNDC3B, 822 different studies on FNDC3B have been included in Oncomine database. Among them, 11 studies indicated the upregulation of FNDC3B and four studies the downregulation in brain and CNS cancers (**Figure 2A**). Then, we evaluated the differentially expressed level of FNDC3B in TCGA pan-cancer data using GEPIA2. Our findings revealed that an elevated expression of FNDC3B was involved in a variety of tumors (**Figure 2C**). In TCGA LGG and GBM cohorts, FNDC3B expression was significantly higher in tumors compared to matched normal tissues (**Figure 2D**). Specifically, FNDC3B expression was 2.71-fold in LGG and 7.16-fold in GBM vs. normal brain tissue. Genetic alterations of FNDC3B in glioma patients were examined using cBioPortal (**Supplementary Figure 1**). Among 6216 samples from 5774 patients in 14 glioma datasets, the overall alteration frequency of FNDC3B gene is 1.5% (71/4774); amplification, mutations, and deep deletions were the most common types of alteration. Due to the low mutation rate, FNDC3B may not be a hypermutation gene in the glioma cohort. In our previous study, we had confirmed that methylation status of TERT was strongly correlated with its expression in hepatocellular carcinoma (HCC) (26). In the present research, the expression of FNDC3B was negatively correlated with FNDC3B DNA methylation; the methylation levels were reduced in the FNDC3B high group based on the TCGA-LGG dataset (**Figures 2E, F**). These results suggested that FNDC3B may be negatively regulated by epigenetic modification and lead to its high expression in glioma samples.

FNDC3B protein expression was explored using HPA database. The immunohistochemistry result revealed upregulated FNDC3B in glioma samples (**Figure 2G**). In human cancer tissues, FNDC3B protein expression in glioma was ranked as the top 12 out of 20 distinct cancer types (**Supplementary Figure 2A**). Furthermore, we detected the FNDC3B mRNA level in human normal tissues using the GTEx database, and FNDC3B expression was mainly found in lung, adipose tissue, thyroid gland, endometrium, and ovary. It is worth noting that the brain displayed the lowest expression levels of global FNDC3B transcript across all normal tissues (**Supplementary Figure 2B**). In addition, we systematically elucidated the expression levels of the FNDC3B in different cancer cells by querying the CCLE database and found that FNDC3B was highly expressed in glioma cell lines (**Supplementary Figure 2C**).

### High expression of FNDC3B predicts poor prognosis of glioma

OncoLnc and GEPIA2 are free online resources and databases for the analysis and visualization of datasets from the TCGA and GTEx projects. To examine the function of FNDC3B on OS in various cancers, we used OncoLnc online tool to perform Cox regression analysis. We found that FNDC3B expression in LGG was ranked first among 21 different cancer types based on the FDR correction (**Table 1**). Moreover, we analyzed the relationships between FNDC3B expression and prognostic values in 33 types of cancer using GEPIA2 databases. As shown in **Supplementary Figure 3**, high FNDC3B expression levels were associated with poorer prognosis of OS and disease-free survival (DFS) in adrenocortical carcinoma (ACC), GBM, kidney Chromophobe (KICH), LGG, liver hepatocellular carcinoma (LIHC), mesothelioma (MESO), and pancreatic adenocarcinoma (PAAD); OS in cervical squamous cell carcinoma and endocervical adenocarcinoma (CESC) and lung adenocarcinoma (LUAD); DFS in colon adenocarcinoma (COAD) and uveal Melanoma (UVM). A high FNDC3B expression was correlated with a better prognosis of OS and DFS in skin cutaneous melanoma (SKCM) as well as OS in acute myeloid leukemia (LAML).

**Table 1:** Cox regression results of FNDC3B among 21 tumor types.

Cancer	Cox Coefficient	P-value	FDR	Rank	Median Expression
LGG	0.82	1.30E-13	2.43E-10	9	324.53
PAAD	0.472	2.00E-05	5.74E-03	57	2620.58
SKCM	-0.242	8.20E-04	1.23E-02	1073	1402.12
KIRP	0.513	1.50E-03	1.33E-02	1827	2117.32
CESC	0.618	2.30E-05	3.41E-02	11	1686.62
LUAD	0.162	3.10E-02	1.55E-01	3320	2465.66
LAML	-0.339	3.00E-03	1.72E-01	260	3845.4
LIHC	0.154	1.10E-01	3.33E-01	5095	1812.82
BLCA	0.113	1.30E-01	3.61E-01	5715	2083.57
BRCA	0.163	7.70E-02	4.29E-01	2968	1715.64
STAD	0.126	1.20E-01	4.70E-01	4198	2732.83
HNSC	0.078	2.60E-01	6.02E-01	7067	2235.09
SARC	0.084	4.40E-01	7.07E-01	10003	2106.74
KIRC	0.031	7.10E-01	7.98E-01	14796	3396.2
GBM	0.137	1.70E-01	8.17E-01	3379	862.45
COAD	-0.03	7.70E-01	9.27E-01	13553	1591.87
LUSC	-0.019	7.80E-01	9.50E-01	13845	2926.55
UCEC	-0.159	1.40E-01	9.64E-01	2309	2368.54
OV	-0.019	8.00E-01	9.65E-01	13931	2115.89
ESCA	-0.06	6.40E-01	9.84E-01	10805	3323.64
READ	-0.067	7.50E-01	9.86E-01	12457	1657.46

To assess the prognostic significance of FNDC3B in glioma patients from TCGA and CGGA, samples were first split into two groups according to the median expression of FNDC3B for each dataset. Among pan-glioma, LGG, and GBM in the TCGA datasets, patients with higher FNDC3B levels presented shorter OS and DFS (**Figure 2H**) compared to patients expressing low levels of FNDC3B. Similarly, high FNDC3B expression was significantly associated with poor prognosis in all the three CGGA datasets (seq_325, seq_693, array_301) (**Supplementary Figure 4**). In particular, highly expressed FNDC3B was significantly related to reduced DFS in GBM (p<0.001), however, just marginally correlated with worse OS (P<0.05). We postulate that the less obvious but significant results of GBM may be due to the insufficient statistical power of a small sample size. Furthermore, we investigated the associations between FNDC3B expression and clinical characteristics, such as age, gender, grade, and isocitrate dehydrogenase (IDH) status. FNDC3B expression was higher in high-grade and IDH wildtype patients; there was no significant difference between age and gender based on the TCGA datasets (**Figure 2I**). Our findings revealed that higher FNDC3B expression is closely correlated with the malignant clinical characters of gliomas.

Subsequently, univariate and multivariate Cox regression analyses were performed to identify whether FNDC3B expression represented an independent prognostic factor. Univariate Cox analysis showed that FNDC3B (HR = 1.64; 95% CI = 1.50-1.80; P < 0.001), grade (HR = 3.37; 95% CI = 2.28-4.98; P < 0.001) and age (HR = 1.06; 95% CI = 1.04-1.07; P < 0.001) were high-risk factors, and IDH mutation (HR = 0.18; 95% CI = 0.07-0.48; P < 0.001) was a low-risk factor (**Table 2**). In multivariate Cox regression analysis, FNDC3B was independently associated with overall survival, suggesting it could be an independent prognostic biomarker for glioma (HR = 1.72; 95% CI = 1.07-2.80; P < 0.05). In addition, age may also be an independent prognostic factor (**Table 2** and **Figure 2J**).

**Table 2:** Univariate and multivariate cox regression analyses of prognostic factors in 416 cases of low-grade glioma (LGG).

Parameter	Univariate analysis		Multivariate analysis	
		HR (95% CI)	P-Value	HR (95% CI)	P-Value
Age (continuous, years)	1.06 (1.04-1.07)	**<0.001**	1.06 (1.01-1.10)	**0.011**
Gender (ref. Female)	1.11 (0.78-1.58)	0.574	2.01 (0.71-5.80)	0.184
Grade (ref. WHO II)	3.37 (2.28-4.98)	**<0.001**	0.85 (0.25-2.9)	0.795
IDH_mutation (ref. Wildtype)	0.18 (0.07-0.48)	**<0.001**	0.40 (0.12-1.40)	0.142
FNDC3B (continuous)	1.64 (1.50-1.80)	**<0.001**	1.72 (1.07-2.80)	**0.026**

Bold values means p<0.05.

### Construction and validation of a prognostic nomogram

**Figure 3:**
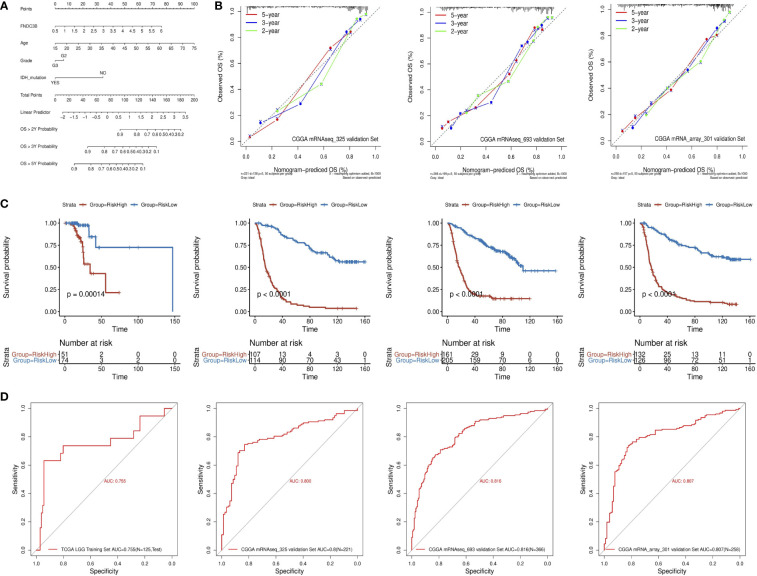
Construction and validation of the prognostic nomogram. **(A)** Prognostic nomogram for TCGA low-grade glioma (LGG) dataset. According to four variables (FNDC3B expression level, age, grade, and IDH status) in the model, four corresponding ‘points’ values can be obtained, and the ‘total points’ can be calculated by summing them. Therefore, the 2-/3-/5-year overall survival (OS) rate of patients can be predicted. **(B)** The calibration curves for predicting 2-/3-/5-year OS in the CGGA_325, CGGA_693, and CGGA_301 validation datasets, respectively. The nomogram-predicted probability of survival and actual survival are plotted on the x- and y-axes, respectively. The diagonal line represents a perfect prediction. **(C)** K-M curve of high-risk (red) and low-risk (blue) for TCGA low-grade glioma (LGG) training, CGGA_325, CGGA_693, and CGGA_301 validation datasets. **(D)** ROC curves for the risk score in the TCGA LGG training, CGGA_325, CGGA_693, and CGGA_301 validation datasets. TCGA, The Cancer Genome Atlas; CGGA, Chinese Glioma Genome Atlas.

A quantitative prognostic nomogram model to predict individual survival chances was established based on the TCGA-LGG dataset using Cox regression (**Figure 3A**). According to the stepwise Cox multivariate regression analysis, age, grade, IDH status, and FNDC3B expression were features that were included in the nomogram, and the risk scores were calculated based on that model. The concordance index (C-index) for OS prediction was 0.775, indicating high predictive performance of the model. A calibration curve was implemented to reflect the degree of consistency between the predicted risk and actual occurrence risk, and it can be used to estimate the accuracy of the model in predicting the probability of an individual outcome in the future (27). In our study, the calibration curve showed acceptable agreement between nomogram-predicted and observed 2-, 3- and 5-year OS in the CGGA_325, CGGA_693, and CGGA_301 validation cohorts (**Figure 3B**).

The patients in the training dataset were divided into high-risk and low-risk groups according to the positive and negative values of the risk score, respectively. The K-M survival curve showed a good discriminating ability of the nomogram (P = 1.4e-4) (**Figure 3C**). Moreover, the area under the ROC curve for OS was 0.755, indicating a reliable predictive ability in the TCGA LGG dataset (**Figure 3D**). The CGGA_325, CGGA_693, and CGGA_301 datasets were used to validate the performance of the nomogram. A risk score for each patient was generated by the same method. Consistently, the patients in the high-risk group had a notably poorer prognosis in all the three validation datasets (p < 0.0001) (**Figure 3C**). The area under the curve (AUC) for CGGA_325, CGGA_693, and CGGA_301 OS was 0.8, 0.816, and 0.807, respectively (**Figure 3D**). In conclusion, these results indicated that the nomogram had adequate performance in predicting the OS of glioma patients.

### DEGs identification and weighted co-expression network construction based on FNDC3B expression

**Figure 4:**
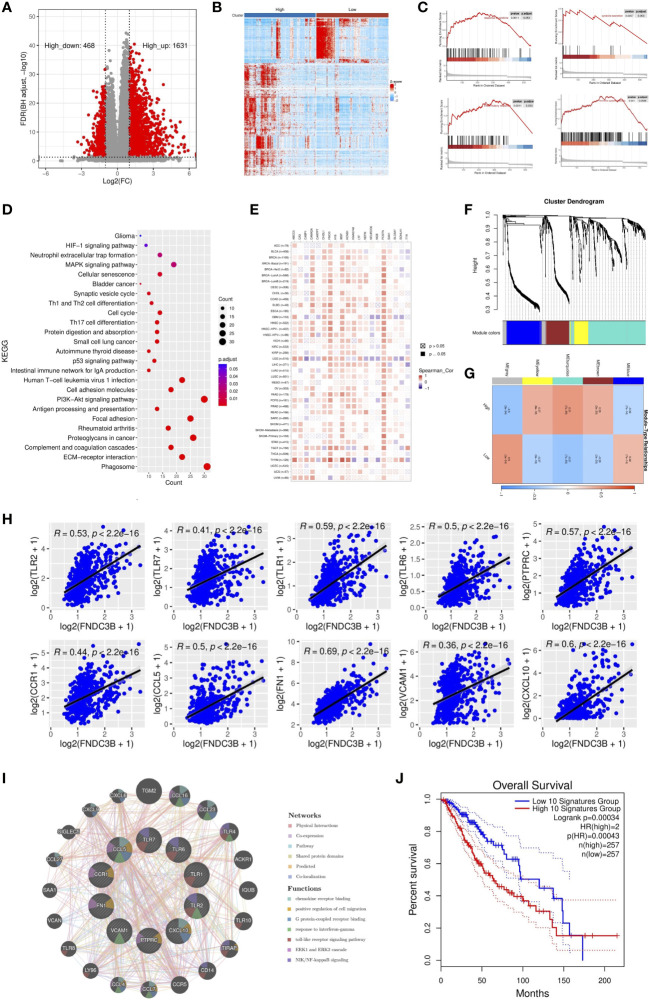
Identification and enrichment analysis of differentially expressed genes (DEGs) in TCGA low-grade glioma (LGG). **(A)** Volcano plot of all DEGs. **(B)** Heatmap of the 547 filtered DEGs. **(C)** Gene set enrichment analysis (GSEA) showed that FNDC3B is involved in the tumor immune microenvironment. **(D)** Kyoto encyclopedia of genes and genomes (KEGG) pathway analyses of DEGs. **(E)** The correlation heatmap between FNDC3B with 20 DEGs in pan-cancer. TCGA: The Cancer Genome Atlas. **(F)** Co-expression network constructed with weighted gene co-expression network analysis (WGCNA), hierarchical clustering tree for DEGs based on a dissimilarity measure (1-TOM), genes with similar expression patterns were merged into the same module. **(G)** Correlation between modules and FNDC3B expression. The upper number in each grid represents the correlation coefficient of each module, and the lower number is the corresponding P-value. **(H)** Relationship of the expression level between the top 10 hub genes and FNDC3B. **(I)** GeneMANIA database analysis shows the interaction network among hub genes. Each node represents a gene. The node size displays the strength of interactions. The line color indicates the types of interactions and the node color represents the possible functions of each gene. **(J)** The survival times of TCGA low-grade glioma (LGG) patients with the highest and lowest expression of the top 10 hub genes were compared.

To further elucidate the role of FNDC3B expression in the glioma microenvironment, the median expression value was used to create a categorical variable for the TCGA-LGG cohort. The DEGs between the two groups were detected using Wilcoxon test. After setting FDR < 0.05 and fold change ≥ 2 in either direction, a total of 2,099 DEGs, including 1,631 upregulated and 468 downregulated genes were screened out in FNDC3B highly expressed group compared to the low expressed group (**Figure 4A**). In order to obtain more reliable results, 670 DEGs were filtered after setting the mean expression value to 1 for the raw DEGs (**Figure 4B**). GSEA was conducted to assess the potential functions of these DEGs. Our results suggested that various immune-related gene signatures were enriched in LGG samples, such as response to cytokine, cytokine secretion, immune system process, and inflammatory response (**Figure 4C**). According to the KEGG analysis, DEGs were mainly concentrated in PI3K-Akt, p53, and MAPK signaling pathways (**Figure 4D**). We used TIMER2.0 to explore the relationship between FNDC3B with the top 10 most significantly up or downregulated DEGs in pan-cancer. The corresponding heatmap showed that the correlation was consistent with the gene expression direction of the 20 DEGs only for LGG (**Figure 4E**).

WGCNA was applied to build a co-expression network based on the 2,099 DEGs. Before constructing the co-expression network, we screened the DEGs by setting expression value larger than 1 in at least 10% of all the samples, and the average level at least 0.5. Finally, 673 DEGs were filtered and selected for subsequent analysis. Power eight was chosen as the appropriate soft threshold because it was the first value to make the degree of independence reach 0.90 and the corresponding average connectivity was close to zero. A total of five gene modules were excavated (genes in the grey module that were not co-expressed; **Figure 4F**). As shown in **Figure 4G**, the turquoise module was the most relevant in FNDC3B expression level (R = 0.51, p = 7e-35). Therefore, the 282 hub genes included in the turquoise module were extracted for further analysis. GO enrichment analyses of these genes indicated that the response to interferon-gamma, neutrophil activation and degranulation, regulation of immune effector process, antigen processing and presentation, T cell activation, leukocyte migration, lymphocyte proliferation, mononuclear cell proliferation, interleukin-8 production and acute inflammatory response was related to FNDC3B-mediated immune events. The top 30 GO items, as ranked by their P-values, are shown in **Supplementary Figure 5**. A total of 210 nodes and 1,061 edges were mapped for the turquoise module genes in the PPI network (**Supplementary Figure 6**). Using CytoHubba in Cytoscape plug-in, we selected the top 10 genes ranked by the MCC method as hub genes, including TLR2, TLR7, PTPRC (CD45), CCR1, CCL5, TLR1, FN1, VCAM1 (CD106), CXCL10, and TLR6. They were immune-related genes and positively correlated with FNDC3B (R > 0.3 and P < 0.0001; **Figure 4H**).

A gene-gene interaction network for the 10 hub genes was built, and their functions were analyzed through the GeneMANIA database (**Figure 4I**). We found that toll-like receptor signaling pathways, ERK1 and ERK2 cascade and NIK/NF-kappaB signaling, were enriched in LGG. Then, OncoLnc online tool was applied to investigate the function of these hub genes on OS of LGG. The results showed that all hub genes were independent risk factors for evaluating OS (**Table 3**), and the K-M curves based on GEPIA2 displayed that higher expression of these genes predicted shorter OS in LGG (**Supplementary Figure 7**). Furthermore, the gene expression analysis of the LGG samples from TCGA showed that the combined expression of the 10 hub genes had a significant effect on overall survival (**Figure 4J**).

**Table 3:** Cox regression results of 10 hub genes in low-grade glioma (LGG).

Gene	Cox coefficient	P-value	FDR
TLR2	0.400	7.30E-06	6.01E-05
TLR7	0.334	2.80E-04	1.18E-03
PTPRC	0.439	2.20E-06	2.30E-05
CCR1	0.306	5.90E-04	2.24E-03
CCL5	0.190	4.90E-02	9.17E-02
TLR1	0.444	2.90E-06	2.88E-05
FN1	0.309	1.20E-03	4.06E-03
VCAM1	0.553	2.40E-09	1.29E-07
CXCL10	0.373	6.40E-05	3.51E-04
TLR6	0.433	3.90E-06	3.65E-05

### Relationship between FNDC3B expression and tumor immune infiltrates

**Figure 5:**
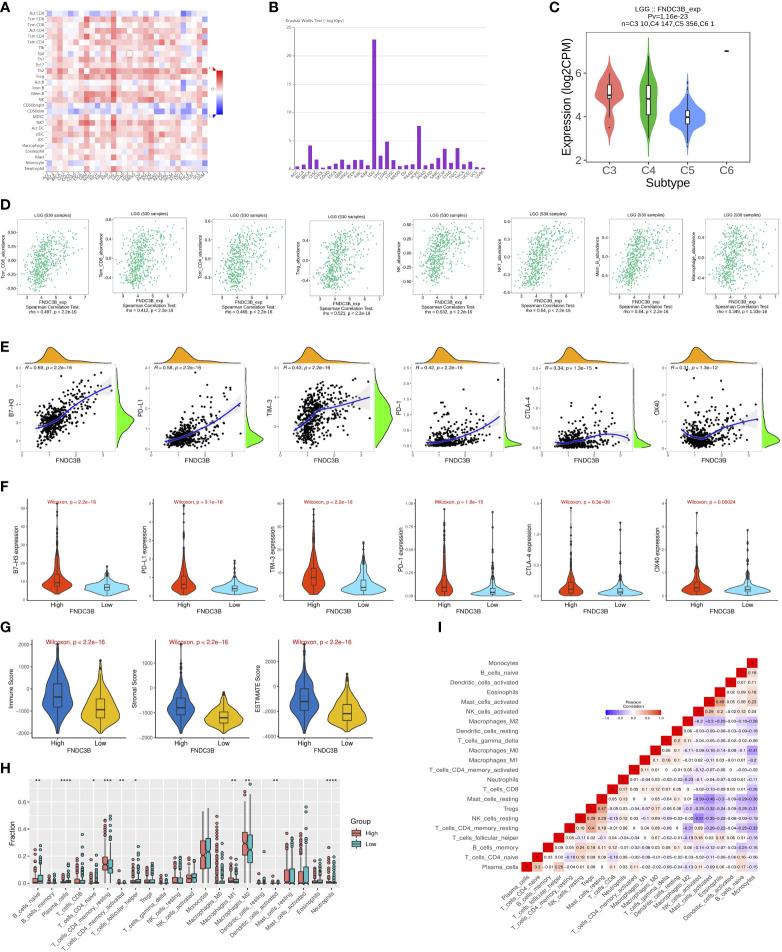
Correlation of FNDC3B expression with immune infiltration level in pan-cancer and TCGA low-grade glioma (LGG). **(A)** The landscape of correlation between FNDC3B expression and tumor-infiltrating lymphocytes (TILs) in pan-cancer (red is positive correlated and blue is negatively correlated). **(B)** Associations between FNDC3B expression and immune subtypes across human cancers. **(C)** Correlation of FNDC3B expression and immune subtypes in low-grade glioma (LGG). C3: inflammatory; C4: lymphocyte depleted; C5: immunologically quiet; C6: TGF-b dominant. **(D)** FNDC3B expression was positively closely related with infiltrating levels of central memory CD8 T cells, effector memory CD8 T cells, central memory CD4 T cells, regulatory T cells, natural killer cells, natural killer T cells, memory B cells, and M1 and M2 macrophages in LGG and glioblastoma multiforme (GBM). **(E)** The correlation between FNDC3B and immune checkpoint molecules (B7-H3, PD-L1, TIM-3, PD-1, CTLA-4, and OX40). **(F)** Different expression levels of the six immune checkpoint genes in the high and low FNDC3B expression groups in TCGA LGG samples. **(G)** Comparison of immune, stromal, and ESTIMATE scores between the FNDCB high- and low-expression groups. **(H)** Different proportions of 22 subtypes of immune cells in the FNDCB high- and low-expression groups in TCGA LGG dataset by CIBERSORTx. The proportions of naive B cells (P < 0.01), plasma cells (P < 0.0001), naive CD4 T cells (P < 0.05), resting memory CD4 T cells (P < 0.001), activated memory CD4 T cells (P < 0.01), follicular helper T cells (P < 0.05), macrophages M1 (P < 0.01), macrophages M2 (P < 0.01), dendritic cells activated (P < 0.01), neutrophils (P < 0.0001). *P < 0.05, **P < 0.01, ***P < 0.001, ****P < 0.0001. **(I)** Correlation matrix heatmap of 22 immune infiltration cells in LGG samples.

Since previous studies have reported that TILs are independent predictors in cancers (28, 29), we used TISIDB database to infer the correlations between the expression of FNDC3B and the abundance of 27 types of TILs across TCGA pan-cancers. As shown in **Figure 5A**, FNDC3B expression was positively correlated with TILs in several human cancer types, especially in LGG. Moreover, we investigated the associations between FNDC3B expression and immune subtypes across human cancers, and the landscape of correlations between FNDC3B expression and immune subtypes in different types of cancer (**Figure 5B**). Among all cancer types, LGG showed the most significant results *via* Kruskal-Wallis test (p = 1.26e-23). In TISIDB, we further analyzed FNDC3B expression in different immune subtypes of LGG. We found FNDC3B was mainly expressed in three types, including C3 (inflammatory type), C4 (lymphocyte depleted type), and C5 (immunologically quiet type). FNDC3B expression was the highest in the C3 (inflammatory) type and the lowest in the C5 (immunologically quiet) type (**Figure 5C**). These results indicated that FNDC3B may play an important role in immune infiltration in glioma. Notably, FNDC3B expression was correlated with the abundance of central memory CD8 T cells (r = 0.497, p < 2.2e-16), effector memory CD8 T cells (r = 0.412, p < 2.2e-16), central memory CD4 T cells (r = 0.468, p < 2.2e-16), regulatory T cells (r = 0.521, p < 2.2e-16), natural killer (NK) cells (r = 0.532, p < 2.2e-16), natural killer T (NKT) cells (r = 0.64, p < 2.2e-16), memory B cells (r = 0.64, p < 2.2e-16), and macrophages (r = 0.349, p < 2.2e-16) in LGG (**Figure 5D**). The positive correlations between FNDC3B expression and TILs were also observed in GBM.

We then assessed the relationships between FNDC3B and eight genes previously reported to be targets of immune checkpoint inhibitors, including CD274 (PD-L1), PDCD1 (PD-1), CD152 (CTLA-4), CD276 (B7-H3), HAVCR2 (TIM-3), CD223 (LAG-3), TNFRSF4 (OX40), and VTCN1. There were significant positive correlations between FNDC3B with B7-H3 (R = 0.69, p < 2.2e-16), PD-L1 (R = 0.58, p < 2.2e-16), TIM-3 (R = 0.43, p < 2.2e-16), PD-1 (R = 0.42, p < 2.2e-16), CTLA-4 (R = 0.34, p = 1.3e-15), and OX40 (R = 0.31, p = 1.3e-12) (**Figure 5E**). Thus, the six genes were significantly upregulated in the FNDC3B high group compared with the low group (**Figure 5F**). In summary, these results suggested that FNDC3B was correlated with clinically relevant immune checkpoint molecules in glioma.

Subsequently, to investigate whether FNDC3B expression was correlated with immune infiltration patterns in LGG, we compared the degree of immune cell infiltration between high and low expression groups using the ESTIMATE algorithm. The immune, stromal, and ESTIMATE scores were higher in the high-expression group than in the low-expression group (**Figure 5G**). Furthermore, we explored the proportions of 22 types of immune cells for LGG using CIBERSORTx to acquire a deeper understanding of the relationship between FNDC3B expression and tumor immune infiltrates. Among the 529 TCGA-LGG samples, 265 samples were in the high expression group and 264 samples in the low expression group. **Figure 5H** shows the differences in the proportions of the 22 subpopulations of immune cells in these two groups. Naive B cells, plasma cells, naive CD4 T cells, resting memory CD4 T cells, activated memory CD4 T cells, follicular helper T cells (Tfh), M1 and M2 macrophages, activated dendritic cells, and neutrophils were the main immune cells affected by FNDC3B expression. Among them, there were more proportions of resting memory CD4 T cells (p < 0.001), activated memory CD4 T cells (p < 0.01), M1 macrophages (p < 0.01), M2 macrophages (p < 0.01), dendritic cells activated (p < 0.01), and neutrophils (p < 0.0001) in the high expression group. In contrast, the proportions of naive B cells (p < 0.01), plasma cells (p < 0.0001), naive CD4 T cells (p < 0.05), and follicular helper T cells (p < 0.05) were lower in the high expression group compared with the low expression group. The correlation matrix heatmap of 22 immune infiltration cells in LGG samples was shown in **Figure 5I**. Moreover, we analyzed the association between FNDC3B expression and gene markers of various TILs, including B cell, plasma cells, T cell, CD4+ T cell, Tfh, M1 and M2 macrophages, dendritic cells, and neutrophils (**Table 4**). Overall, FNDC3B expression was strongly positively correlated with gene markers of B cells, T cells, M1 and M2 macrophages, dendritic cells, and neutrophils for TCGA-LGG and TCGA-GBM.

**Table 4:** Correlation analysis between FNDC3B expression and gene markers of immune cells in Gene Expression Profiling Interactive Analysis 2 (GEPIA2).

Immune cell types	Gene markers	LGG		GBM	
				Cor	P	Cor	P
B cells	CD2	0.42	***	0.3	***
	CD74	0.48	***	0.31	***
	CD27	0.24	***	0.3	***
Plasma cells	SPAG4	0.3	***	0.29	***
	PDK1	0.37	***	0.2	**
	MAST1	-0.31	***	−0.27	***
	MANEA	0.64	***	0.2	*
T cells	CD2	0.42	***	0.3	***
	CD3E	0.38	***	0.32	***
	CD3D	0.37	***	0.21	**
CD4+ T cells	CD4	0.47	***	0.42	***
Tfh	BCL6	0.16	***	0.28	***
	CD84	0.47	***	0.32	***
	IL6R	0.46	***	0.4	***
	IL21	0.22	***	0.19	*
M1 Macrophage	CD80	0.36	***	0.28	***
	IRF5	0.41	***	0.26	***
	IL6	0.19	***	0.42	***
	CD64	0.24	***	0.21	**
M2 Macrophage	CD163	0.4	***	0.43	***
	CD206	0.12	**	0.57	***
	VSIG4	0.34	***	0.3	***
	MS4A4A	0.35	***	0.36	***
Dendritic cell	HLA-DPB1	0.47	***	0.3	***
	HLA-DQB1	0.36	***	0.27	***
	HLA-DRA	0.52	***	0.24	**
	HLA-DPA1	0.47	***	0.27	***
	CD8A	0.43	***	0.25	**
	CD141	0.35	***	0.6	***
	NRP1	0.63	***	0.81	***
Neutrophils	CCR1	0.45	***	0.41	***
	CD11B	0.46	***	0.56	***
	CCR7	0.25	***	0.3	***
	SLC1A5	0.42	***	0.43	***
	CXCR2	0.38	***	0.22	**

*P < 0.05; **P < 0.01; ***P < 0.001.

## Discussion

Glioma is the most common primary intracranial neoplasm, accounting for approximately 80% of malignant brain tumors. The limitations of classical treatments lead to a poor OS (30). New progress in brain tumor research suggests that immunotherapy is a powerful tool for the treatment of gliomas (31–33). Therefore, the identification of novel effective biomarkers for early diagnosis and promising immune-related therapeutic targets for glioma patients has become imperative in clinical practice. Recently, several studies have reported that FNDC3B is an oncogene in various cancers, including glioma (18). To our knowledge, the expression pattern and biological function of FNDC3B in glioma have not been studied in detail, and its possible prognostic value in glioma remains to be explored. Based on the integrated machine learning methods, this is the first report to comprehensively analyze FNDC3B expression profiles and its correlation with immune infiltrates in gliomas.

In this study, we identified that FNDC3B was highly expressed in glioma tissues by mining multiple databases, and the expression levels of FNDC3B increased with the level of the malignant degree, which was also confirmed in another study (16). These results suggested that FNDC3B could serve as a promising molecular marker for predicting the degree of malignancy in brain glioma. K-M plots indicated that patients with high FNDC3B expression had worse OS and DFS than those with low expression in gliomas. Furthermore, univariate and multivariate Cox analysis demonstrated a positive correlation between FNDC3B expression and poor prognosis of patients with glioma. To further apply FNDC3B in clinical treatments, a prognostic nomogram for personalized prediction was constructed, integrating the FNDC3B expression level with significant clinical parameters (age, grade, and IDH status). The C-index, AUC value, and calibration curve for TCGA training and three CGGA validation datasets showed that our nomogram was reliable and performed adequately. In general, we identified and validated the expression level of FNDC3B as a useful and independent prognostic biomarker for glioma. In the present study, we found that the expression of FNDC3B was negatively correlated with its DNA methylation and CNV was the most common type of alteration for FNDC3B gene. Compared with single-omics, multi-omics approaches can provide more deep insights on molecular changes for cancer subtyping (34). A recent study identified subtype-specific signatures *via* a computational framework for analyzing multi-omics profiles and patient survival and confirmed that subtype-specific signatures could be more feasible in the clinical practice (35). By combining multidimensional genomic measurements, a higher resolution of prognostic signatures will be available for different glioma subtypes in the future. Moreover, methylation (5mC) in cell-free DNA (cfDNA) have been widely observed in human diseases, regions with consistently altered 5mC levels for FNDC3B in circulating cfDNA during progression from low-grade glioma to glioblastoma could be used as markers for development of minimally invasive screening of early diagnosis and surveillance (36, 37).

To further investigate the functions of FNDC3B in glioma, we performed GSEA analysis using DEGs based on TCGA LGG data. The results showed that multiple immune-related pathways were enriched in the FNDC3B high-expression group, such as cellular response to cytokine stimulus, inflammatory response, and immune system process. In the KEGG analysis, PI3K-Akt, p53 and MAPK signaling pathways participated in tumor development. We found that the expression of FNDC3B correlated with that of multiple T cell markers (Th1, Th2, and Th17) in LGG. This suggested that FNDC3B may be involved in the regulation of T cell response in glioma. In addition, WGCNA was performed on the DEGs to find more valuable clues. Finally, 10 upregulated genes were identified as hub genes, including TLR2, TLR7, PTPRC, CCR1, CCL5, TLR1, FN1, VCAM1, CXCL10, and TLR6. Previous studies reported that T cells have the ability to directly recognize danger signals through the expression of toll-like receptors (TLRs) (38); interactions between CCR1 and CCL5 contribute to T-cell activation (39), and CXCL10 is usually considered to be a pro-inflammatory chemokine that enhances recruitment of CD8+ and Th1-type CD4+ effector T cells to infected or inflamed nonlymphoid tissues (40). FN1 can promote integrin β1 ubiquitination and degradation and its expression may be upregulated by the hyperactivation of ERK1/2 (41), considered to be critical mediators for T cell functions (42). VCAM1 induces T-cell antigen receptor-dependent activation of CD4+ T lymphocytes (43), and PTPRC is a well-known positive regulator of T-cell receptor signaling (44). Overall, these findings highlight the ability of FNDC3B to potentially regulate T cell responses in LGG.

TILs are independent predictors in cancers (29). Our findings showed that FNDC3B was strongly positively correlated with immune infiltration in LGG and GBM among all cancer types in the database, especially in the cytotoxic T cells and anti-tumor associated immune cells, such as central memory CD8 T cell, effector memory CD8 T cell, central memory CD4 T cell, regulatory T cell (Treg), natural killer cell (NK), natural killer T cell (NKT), memory B cell, and macrophage. The comparative analysis of FNDC3B gene expression in different immune subtypes in LGG suggested that FNDC3B may be strongly linked to immunological properties in the tumor microenvironment. A previous study reported that the immune microenvironment affected the gene expression of tumor tissues, and the degree of stromal and immune cell infiltration influenced prognosis (45). In our study, the FNDC3B high-expression group displayed higher values for immune, stromal, and ESTIMATE scores than the FNDC3B low-expression group, indicating that the high expression level of FNDC3B is positively related to immune infiltration in gliomas. Moreover, consistent with the TISIDB results, we found that memory CD4 T cell, macrophages M1 and M2, and neutrophils were enriched in the FNDC3B high group based on CIBERSORT analysis. Additionally, a relatively strong correlation between FNDC3B expression and gene markers of T cells, CD4+ T cell, follicular helper T cells, and dendritic cells indicated the potential role of FNDC3B in regulating T cell function in LGG and GBM.

The FNDC3B expression was positively correlated with genes of immune checkpoints, suggesting that FNDC3B could be a regulatory factor of various immune checkpoints in glioma. The correlation analysis showed that FNDC3B was mostly positively correlated with B7-H3, which was associated with a suppressive effect on T-cell activities in various tumors (46). In recent years, studies have shown that B7-H3 is a promising novel target for glioma immunotherapy (47, 48). Nehama and colleagues reported that B7-H3 is highly expressed in more than 70% of GBM samples and that B7-H3-redirected chimeric antigen receptor T (CAR-T) cells can effectively control tumor growth (31). Currently, tumor immunotherapy has attracted great attention. Hence, our findings indicated that whether we can participate in the immune checkpoint by inhibiting the expression of FNDC3B, and can FNDC3B be served as a powerful immune checkpoint blockade combination therapy to increase efficacy and reduce side effects? More investigations are required to get a full description and understanding of the mechanisms in the future, this study provide a new insight for further exploration of the molecular mechanisms.

There are several limitations in the current research. First, it was mainly based on online public databases and computational methods. Nevertheless, integrated machine learning algorithms strengthen the conclusion of this study. Second, more investigations are needed to identify the expression and function of FNDC3B as well as their correlations with immune cell infiltration; thus, further clinical and experimental studies in the laboratory are required for verifying its role in glioma.

In conclusion, our comprehensive analysis revealed that FNDC3B was upregulated in glioma, while increased FNDC3B expression predicted an unfavorable prognosis. Moreover, FNDC3B is associated with the infiltration of various immune cells, and it may play a vital role in the tumor immune microenvironment of glioma. Therefore, we reported that FNDC3B is a possible prognostic biomarker and an immune-related therapeutic target for glioma, which will be useful for clinical applications.

## Data availability statement

The original contributions presented in the study are included in the article/**Supplementary Material**. Further inquiries can be directed to the corresponding authors.

## Ethics statement

The studies involving human participants were reviewed and approved by The Ethics Committee of the Shanghai Jiao Tong University Affiliated Sixth People’s Hospital. Written informed consent for participation was not required for this study in accordance with the national legislation and the institutional requirements.

## Author contributions

Conception and design: XW and HZ. Acquisition and analysis of the data: XW, YH, SL, and HZ. Draft and revision of the manuscript: XW and HZ. All authors have read and approved the submitted version.

## Funding

The current study was supported by the National NaturalScience Foundation of China (No. 81800708), the ShanghaiSixth People’s Hospital Grant (No. ynhg202204 andynqn202105), the China Postdoctoral Science Foundation Grant (No. 2019M660088 and 2020T13408).

## Acknowledgments

We greatly appreciate for the analytic data provided by the TCGA and CGGA databases. The draft was preprinted at https://www.researchsquare.com/article/rs-1102200/v1 2021.

## Conflict of interest

The authors declare that the research was conducted in the absence of any commercial or financial relationships that could be construed as a potential conflict of interest.

## Publisher’s note

All claims expressed in this article are solely those of the authors and do not necessarily represent those of their affiliated organizations, or those of the publisher, the editors and the reviewers. Any product that may be evaluated in this article, or claim that may be made by its manufacturer, is not guaranteed or endorsed by the publisher.

## References

[1] Louis DN Perry A Reifenberger G von Deimling A Figarella-Branger D Cavenee WK. The 2016 world health organization classification of tumors of the central nervous system: A summary. Acta Neuropathol (2016) 131(6):803–20. doi: 10.1007/s00401-016-1545-1 27157931

[2] Jhaveri J Liu Y Chowdhary M Buchwald ZS Gillespie TW Olson JJ. Is less more? comparing chemotherapy alone with chemotherapy and radiation for high-risk grade 2 glioma: An analysis of the national cancer data base. Cancer (2018) 124(6):1169–78. doi: 10.1002/cncr.31158 29205287

[3] Zheng H Luo L Zhao W. Factors associated with level III lymph nodes positive and survival analysis of its dissection in patients with breast cancer. Laparoscopic Endoscopic Robotic Surg (2020) 3(2):43–7. doi: 10.1016/j.lers.2020.03.001

[4] Das S Camphausen K Shankavaram U. Cancer-specific immune prognostic signature in solid tumors and its relation to immune checkpoint therapies. Cancers (Basel) (2020) 12(9):2476. doi: 10.3390/cancers12092476 PMC7563367 32882873

[5] Zhang Z Chen L Chen H Zhao J Li K Sun J. Pan-cancer landscape of T-cell exhaustion heterogeneity within the tumor microenvironment revealed a progressive roadmap of hierarchical dysfunction associated with prognosis and therapeutic efficacy. EBioMedicine (2022) 83:104207. doi: 10.1016/j.ebiom.2022.104207 35961204 PMC9382263

[6] Lei J Zhou MH Zhang FC Wu K Liu SW Niu HQ. Interferon regulatory factor transcript levels correlate with clinical outcomes in human glioma. Aging (Albany NY) (2021) 13(8):12086–98. doi: 10.18632/aging.202915 PMC8109055 33902005

[7] Bao S Zhao H Yuan J Fan D Zhang Z Su J. Computational identification of mutator-derived lncRNA signatures of genome instability for improving the clinical outcome of cancers: a case study in breast cancer. Brief Bioinform (2020) 21(5):1742–55. doi: 10.1093/bib/bbz118 31665214

[8] Zhou M Zhang Z Zhao H Bao S Cheng L Sun J. An immune-related six-lncRNA signature to improve prognosis prediction of glioblastoma multiforme. Mol Neurobiol (2018) 55(5):3684–97. doi: 10.1007/s12035-017-0572-9 28527107

[9] Kishimoto K Kato A Osada S Nishizuka M Imagawa M. Fad104, a positive regulator of adipogenesis, negatively regulates osteoblast differentiation. Biochem Biophys Res Commun (2010) 397(2):187–91. doi: 10.1016/j.bbrc.2010.05.077 20493170

[10] Fucci C Resnati M Riva E Perini T Ruggieri E Orfanelli U. The interaction of the tumor suppressor FAM46C with p62 and FNDC3 proteins integrates protein and secretory homeostasis. Cell Rep (2020) 32(12):108162. doi: 10.1016/j.celrep.2020.108162 32966780

[11] Nishizuka M Kishimoto K Kato A Ikawa M Okabe M Sato R. Disruption of the novel gene fad104 causes rapid postnatal death and attenuation of cell proliferation, adhesion, spreading and migration. Exp Cell Res (2009) 315(5):809–19. doi: 10.1016/j.yexcr.2008.12.013 19138685

[12] Chen CF Hsu EC Lin KT Tu PH Chang HW Lin CH. Overlapping high-resolution copy number alterations in cancer genomes identified putative cancer genes in hepatocellular carcinoma. Hepatology (2010) 52(5):1690–701. doi: 10.1002/hep.23847 20799341

[13] Cheng CK Wang AZ Wong THY Wan TSK Cheung JS Raghupathy R. FNDC3B is another novel partner fused to RARA in the t(3;17)(q26;q21) variant of acute promyelocytic leukemia. Blood (2017) 129(19):2705–9. doi: 10.1182/blood-2017-02-767707 28314734

[14] Li Y Yang J Wang H Qiao W Guo Y Zhang S. FNDC3B, targeted by miR-125a-5p and miR-217, promotes the proliferation and invasion of colorectal cancer cells via PI3K/mTOR signaling. Onco Targets Ther (2020) 13:3501–10. doi: 10.2147/OTT.S226520 PMC7201223 32431508

[15] Han B Wang H Zhang J Tian J. FNDC3B is associated with ER stress and poor prognosis in cervical cancer. Oncol Lett (2020) 19(1):406–14. doi: 10.3892/ol.2019.11098 PMC6924122 31897153

[16] Wang GH Wang LY Zhang C Zhang P Wang CH Cheng S. MiR-1225-5p acts as tumor suppressor in glioblastoma via targeting FNDC3B. Open Med (Wars) (2020) 15(1):872–81. doi: 10.1515/med-2020-0156 PMC7712056 33336045

[17] Xu H Hu Y Qiu W. Potential mechanisms of microRNA-129-5p in inhibiting cell processes including viability, proliferation, migration and invasiveness of glioblastoma cells U87 through targeting FNDC3B. BioMed Pharmacother (2017) 87:405–11. doi: 10.1016/j.biopha.2016.12.100 28068630

[18] Wang Z Tang W Yuan J Qiang B Han W Peng X. Integrated analysis of RNA-binding proteins in glioma. Cancers (Basel) (2020) 12(4):892. doi: 10.3390/cancers12040892 PMC7226056 32272554

[19] Rajasagi M Shukla SA Fritsch EF Keskin DB DeLuca D Carmona E. Systematic identification of personal tumor-specific neoantigens in chronic lymphocytic leukemia. Blood (2014) 124(3):453–62. doi: 10.1182/blood-2014-04-567933 PMC4102716 24891321

[20] Sun J Zhang Z Bao S Yan C Hou P Wu N. Identification of tumor immune infiltration-associated lncRNAs for improving prognosis and immunotherapy response of patients with non-small cell lung cancer. J Immunother Cancer (2020) 8(1):e000110. doi: 10.1136/jitc-2019-000110 32041817 PMC7057423

[21] Bao S Hu T Liu J Su J Sun J Ming Y. Genomic instability-derived plasma extracellular vesicle-microRNA signature as a minimally invasive predictor of risk and unfavorable prognosis in breast cancer. J Nanobiotechnol (2021) 19(1):22. doi: 10.1186/s12951-020-00767-3 PMC7802300 33436002

[22] Zhang H Ye J Weng X Liu F He L Zhou D. Comparative transcriptome analysis reveals that the extracellular matrix receptor interaction contributes to the venous metastases of hepatocellular carcinoma. Cancer Genet (2015) 208(10):482–91. doi: 10.1016/j.cancergen.2015.06.002 26271415

[23] Rhodes DR Yu J Shanker K Deshpande N Varambally R Ghosh D. ONCOMINE: a cancer microarray database and integrated data-mining platform. Neoplasia (2004) 6(1):1–6. doi: 10.1016/S1476-5586(04)80047-2 15068665 PMC1635162

[24] Tang Z Li C Kang B Gao G Li C Zhang Z. GEPIA: a web server for cancer and normal gene expression profiling and interactive analyses. Nucleic Acids Res (2017) 45(W1):W98–W102. doi: 10.1093/nar/gkx247 28407145 PMC5570223

[25] Iasonos A Schrag D Raj GV Panageas KS. How to build and interpret a nomogram for cancer prognosis. J Clin Oncol (2008) 26(8):1364–70. doi: 10.1200/JCO.2007.12.9791 18323559

[26] Zhang H Weng X Ye J He L Zhou D Liu Y. Promoter hypermethylation of TERT is associated with hepatocellular carcinoma in the han Chinese population. Clin Res Hepatol Gastroenterol (2015) 39(5):600–9. doi: 10.1016/j.clinre.2015.01.002 25683523

[27] Huang YQ Liang CH He L Tian J Liang CS Chen X. Development and validation of a radiomics nomogram for preoperative prediction of lymph node metastasis in colorectal cancer. J Clin Oncol (2016) 34(18):2157–64. doi: 10.1200/JCO.2015.65.9128 27138577

[28] Ohtani H. Focus on TILs: prognostic significance of tumor infiltrating lymphocytes in human colorectal cancer. Cancer Immun (2007) 7:4. doi: 10.1158/1424-9634.DCL-4.7.1 17311363 PMC2935759

[29] Azimi F Scolyer RA Rumcheva P Moncrieff M Murali R McCarthy SW. Tumor-infiltrating lymphocyte grade is an independent predictor of sentinel lymph node status and survival in patients with cutaneous melanoma. J Clin Oncol (2012) 30(21):2678–83. doi: 10.1200/JCO.2011.37.8539 22711850

[30] Goodenberger ML Jenkins RB. Genetics of adult glioma. Cancer Genet (2012) 205(12):613–21. doi: 10.1016/j.cancergen.2012.10.009 23238284

[31] Nehama D Di Ianni N Musio S Du H Patane M Pollo B. B7-H3-redirected chimeric antigen receptor T cells target glioblastoma and neurospheres. EBioMedicine (2019) 47:33–43. doi: 10.1016/j.ebiom.2019.08.030 31466914 PMC6796553

[32] Zhang H Bi Y Wei Y Liu J Kuerban K Ye L. Blocking wnt/beta-catenin signal amplifies anti-PD-1 therapeutic efficacy by inhibiting tumor growth, migration, and promoting immune infiltration in glioblastomas. Mol Cancer Ther (2021) 20(7):1305–15. doi: 10.1158/1535-7163.MCT-20-0825 34001635

[33] Anghileri E Di Ianni N Paterra R Langella T Zhao J Eoli M. High tumor mutational burden and T-cell activation are associated with long-term response to anti-PD1 therapy in lynch syndrome recurrent glioblastoma patient. Cancer Immunol Immunother (2021) 70(3):831–42. doi: 10.1007/s00262-020-02769-4 PMC10992921 33140187

[34] Sun J Yan C Xu D Zhang Z Li K Li X. Immuno-genomic characterisation of high-grade serous ovarian cancer reveals immune evasion mechanisms and identifies an immunological subtype with a favourable prognosis and improved therapeutic efficacy. Br J Cancer (2022) 126(11):1570–80. doi: 10.1038/s41416-021-01692-4 PMC9130248 35017656

[35] Yu F Quan F Xu J Zhang Y Xie Y Zhang J. Breast cancer prognosis signature: linking risk stratification to disease subtypes. Brief Bioinform (2019) 20(6):2130–40. doi: 10.1093/bib/bby073 30184043

[36] Yu F Li K Li S Liu J Zhang Y Zhou M. CFEA: a cell-free epigenome atlas in human diseases. Nucleic Acids Res (2020) 48(D1):D40–4. doi: 10.1093/nar/gkz715 PMC6943076 31428785

[37] Hlady RA Zhao X Pan X Yang JD Ahmed F Antwi SO. Genome-wide discovery and validation of diagnostic DNA methylation-based biomarkers for hepatocellular cancer detection in circulating cell free DNA. Theranostics (2019) 9(24):7239–50. doi: 10.7150/thno.35573 PMC6831291 31695765

[38] Salerno F Freen-van Heeren JJ Guislain A Nicolet BP Wolkers MC. Costimulation through TLR2 drives polyfunctional CD8(+) T cell responses. J Immunol (2019) 202(3):714–23. doi: 10.4049/jimmunol.1801026 30578304

[39] Choi SW Hildebrandt GC Olkiewicz KM Hanauer DA Chaudhary MN Silva IA. CCR1/CCL5 (RANTES) receptor-ligand interactions modulate allogeneic T-cell responses and graft-versus-host disease following stem-cell transplantation. Blood (2007) 110(9):3447–55. doi: 10.1182/blood-2007-05-087403 PMC2200916 17641205

[40] Peperzak V Veraar EA Xiao Y Babala N Thiadens K Brugmans M. CD8+ T cells produce the chemokine CXCL10 in response to CD27/CD70 costimulation to promote generation of the CD8+ effector T cell pool. J Immunol (2013) 191(6):3025–36. doi: 10.4049/jimmunol.1202222 23940275

[41] Wang Y Li C Shi L Chen X Cui C Huang J. Integrin beta1D deficiency-mediated RyR2 dysfunction contributes to catecholamine-sensitive ventricular tachycardia in arrhythmogenic right ventricular cardiomyopathy. Circulation (2020) 141(18):1477–93. doi: 10.1161/CIRCULATIONAHA.119.043504 PMC7200284 32122157

[42] Crawford TQ Hecht FM Pilcher CD Ndhlovu LC Barbour JD. Activation associated ERK1/2 signaling impairments in CD8+ T cells co-localize with blunted polyclonal and HIV-1 specific effector functions in early untreated HIV-1 infection. PloS One (2013) 8(10):e77412. doi: 10.1371/journal.pone.0077412 24143233 PMC3797111

[43] Damle NK Aruffo A. Vascular cell adhesion molecule 1 induces T-cell antigen receptor-dependent activation of CD4+T lymphocytes. Proc Natl Acad Sci U.S.A. (1991) 88(15):6403–7. doi: 10.1073/pnas.88.15.6403 PMC52093 1713678

[44] Porcu M Kleppe M Gianfelici V Geerdens E De Keersmaecker K Tartaglia M. Mutation of the receptor tyrosine phosphatase PTPRC (CD45) in T-cell acute lymphoblastic leukemia. Blood (2012) 119(19):4476–9. doi: 10.1182/blood-2011-09-379958 22438252

[45] Winslow S Lindquist KE Edsjo A Larsson C. The expression pattern of matrix-producing tumor stroma is of prognostic importance in breast cancer. BMC Cancer (2016) 16(1):841. doi: 10.1186/s12885-016-2864-2 27809802 PMC5095990

[46] Kontos F Michelakos T Kurokawa T Sadagopan A Schwab JH Ferrone CR. B7-H3: An attractive target for antibody-based immunotherapy. Clin Cancer Res (2021) 27(5):1227–35. doi: 10.1158/1078-0432.CCR-20-2584 PMC7925343 33051306

[47] Zhang C Zhang Z Li F Shen Z Qiao Y Li L. Large-Scale analysis reveals the specific clinical and immune features of B7-H3 in glioma. Oncoimmunology (2018) 7(11):e1461304. doi: 10.1080/2162402X.2018.1461304 30377558 PMC6205005

[48] Tang X Zhao S Zhang Y Wang Y Zhang Z Yang M. B7-H3 as a novel CAR-T therapeutic target for glioblastoma. Mol Ther Oncolytics (2019) 14:279–87. doi: 10.1016/j.omto.2019.07.002 PMC6713854 31485480

