# Supplementary material for: Integrated machine learning methods identify FNDC3B as a potential prognostic biomarker and correlated with immune infiltrates in glioma
Source: Front Immunol. 2022 Oct 6;13:1027154. doi: 10.3389/fimmu.2022.1027154 (PMC9582524; doi:10.3389/fimmu.2022.1027154)

Supplementary Figure 1. Genetic alterations of FNDC3B in glioma patients. (A) OncoPrint visual summary of variations on FNDC3B; (B) Summary of genetic alterations for FNDC3B in 14 glioma datasets.

(A)

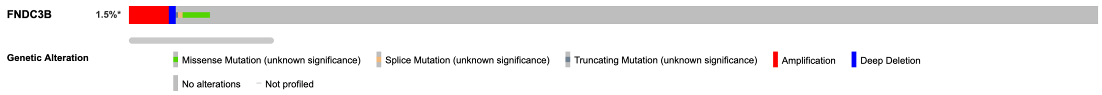

(B)

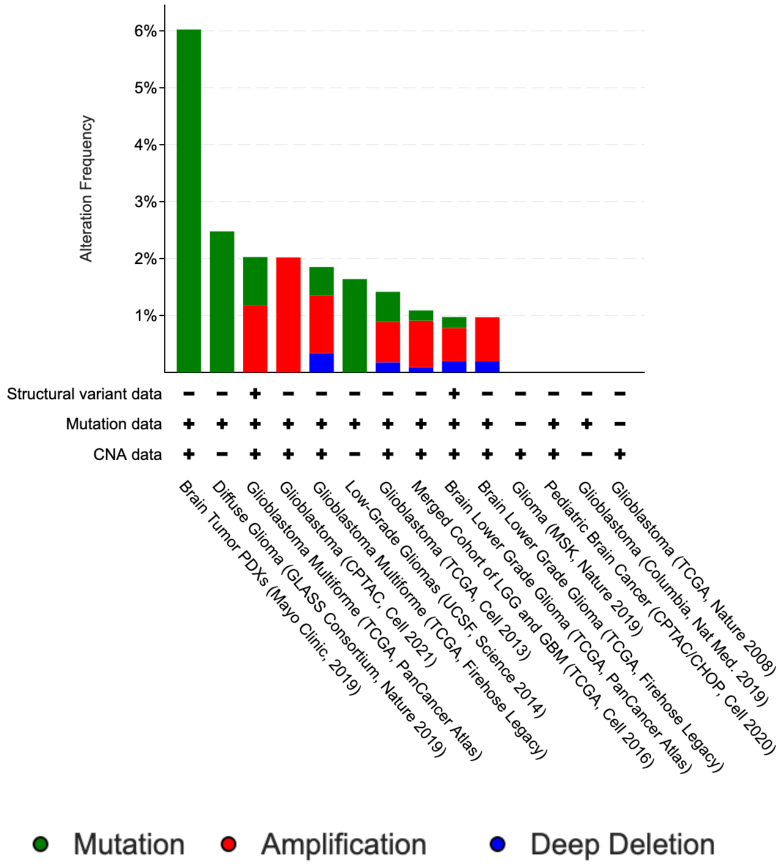

Supplementary Figure 2. FNDC3B expression profiles in human tissues and cancer cell lines. (A) The protein expression profiles of FNDC3B in human cancer tissues. (B) FNDC3B expression profiles in normal human tissues. (C) The expression of FNDC3B in glioma cell lines analyzed by Cancer Cell Line Encyclopedia (CCLE).

(A)

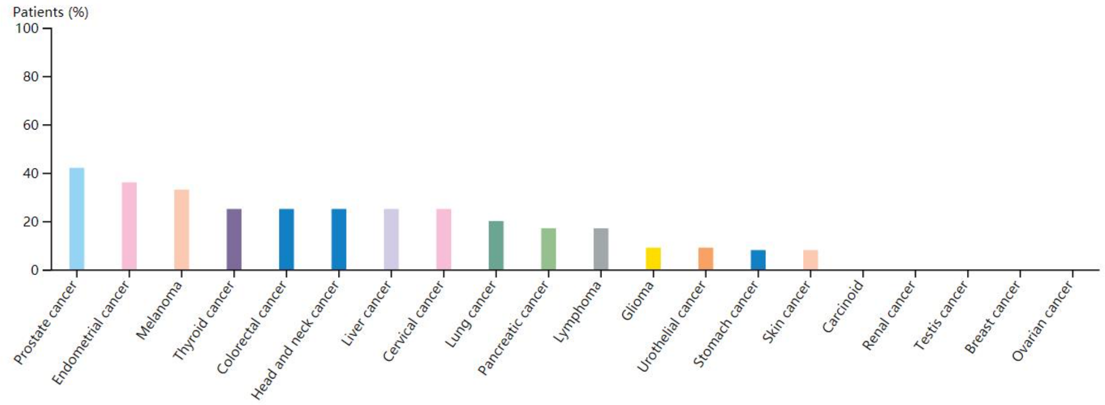

(B)

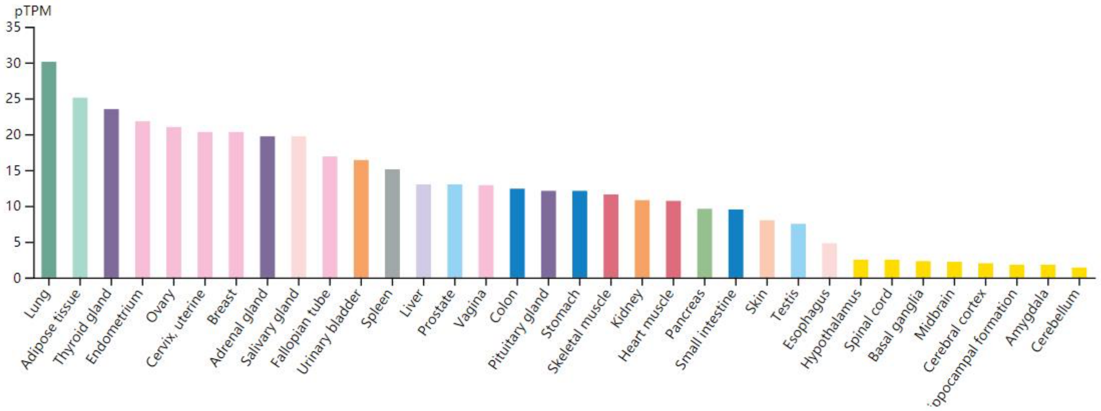

(C)

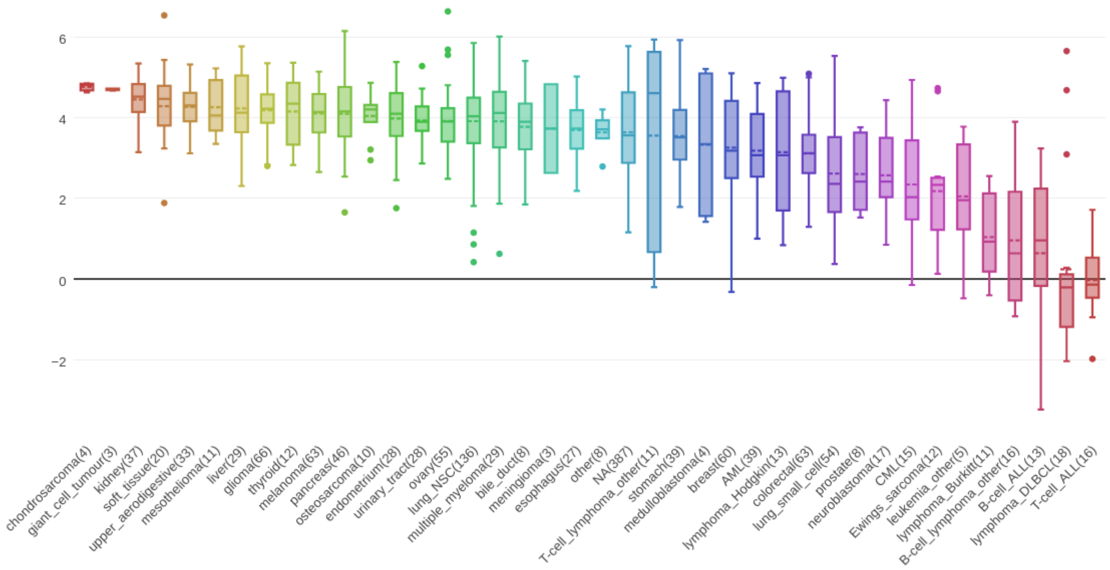

Supplementary Figure 3. Correlation of FNDC3B expression with prognostic values in diverse types of cancer. Overall survival and disease free curves comparing the high and low expression of FNDC3B in (A-B) Adrenocortical carcinoma (ACC), (C-D) Bladder Urothelial Carcinoma (BLCA), (E-F) Breast invasive carcinoma (BRCA), (G-H) Cervical squamous cell carcinoma and endocervical adenocarcinoma (CESC), (I-J) Cholangio carcinoma (CHOL), (K-L) Colon adenocarcinoma (COAD), (M-N) Lymphoid Neoplasm Diffuse Large B-cell Lymphoma (DLBC), (O-P) Esophageal carcinoma (ESCA), (QR) Glioblastoma multiforme (GBM), (S-T) Head and Neck squamous cell carcinoma (HNSC), (U-V) Kidney Chromophobe (KICH), (W-X) Kidney renal clear cell carcinoma (KIRC), (Y-Z) Kidney renal papillary cell carcinoma (KIRP), (AA-AB) Acute Myeloid Leukemia (LAML), (AC-AD) Brain Lower Grade Glioma (LGG), (AE-AF) Liver hepatocellular carcinoma (LIHC), (AG-AH) Lung adenocarcinoma (LUAD), (AI-AJ) Lung squamous cell carcinoma (LUSC), (AK-AL) Mesothelioma (MESO), (AM-AN) Ovarian serous cystadenocarcinoma (OV), (AO-AP) Pancreatic adenocarcinoma (PAAD), (AQ-AR) Pheochromocytoma and Paraganglioma (PCPG), (AS-AT) Prostate adenocarcinoma (PRAD), (AU-AV) Rectum adenocarcinoma (READ), (AW-AX) Sarcoma (SARC), (AY-AZ) Skin Cutaneous Melanoma (SKCM), (BA-BB) Stomach adenocarcinoma (STAD), (BC-BD) Testicular Germ Cell Tumors (TGCT), (BE-BF) Thyroid carcinoma (THCA), (BG-BH) Thymoma (THYM), (BI-BJ) Uterine Corpus Endometrial Carcinoma (UCEC), (BK-BL) Uterine Carcinosarcoma (UCS), (BM-BN) Uveal Melanoma (UVM).

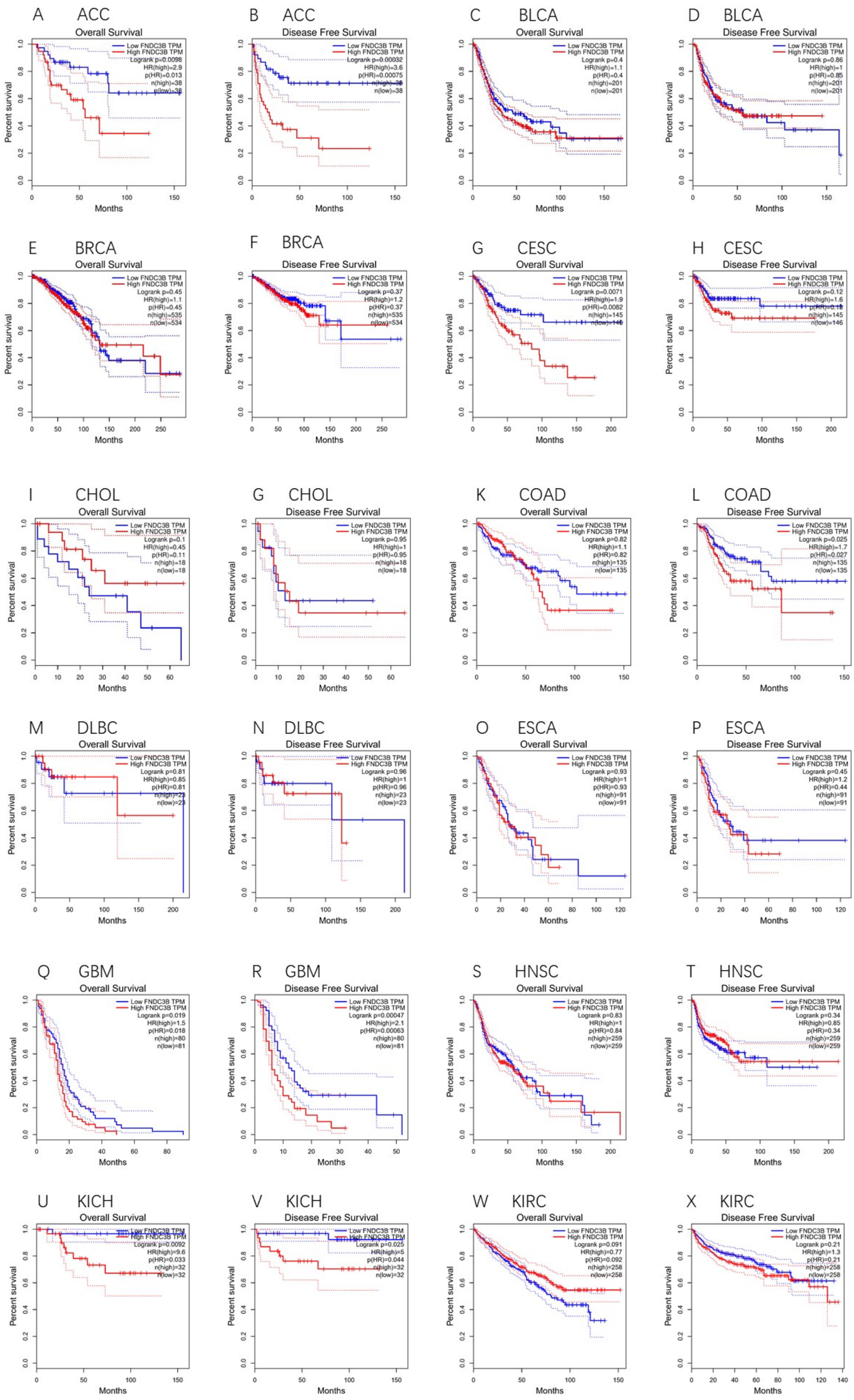
A ACC
Overall Survival
B ACC
Disease Free Survival
C BLCA
Overall Survival
D BLCA
Disease Free Survival
E BRCA
Overall Survival
F BRCA
Disease Free Survival
G CESC
Overall Survival
H CESC
Disease Free Survival
I CHOL
Overall Survival
G CHOL
Disease Free Survival
K COAD
Overall Survival
L COAD
Disease Free Survival
M DLBC
Overall Survival
N DLBC
Disease Free Survival
O ESCA
Overall Survival
P ESCA
Disease Free Survival
Q GBM
Overall Survival
R GBM
Disease Free Survival
S HNSC
Overall Survival
T HNSC
Disease Free Survival
U KICH
Overall Survival
V KICH
Disease Free Survival
W KIRC
Overall Survival
X KIRC
Disease Free Survival
Low FNDC3B TPM
High FNDC3B TPM
Percent survival
Months

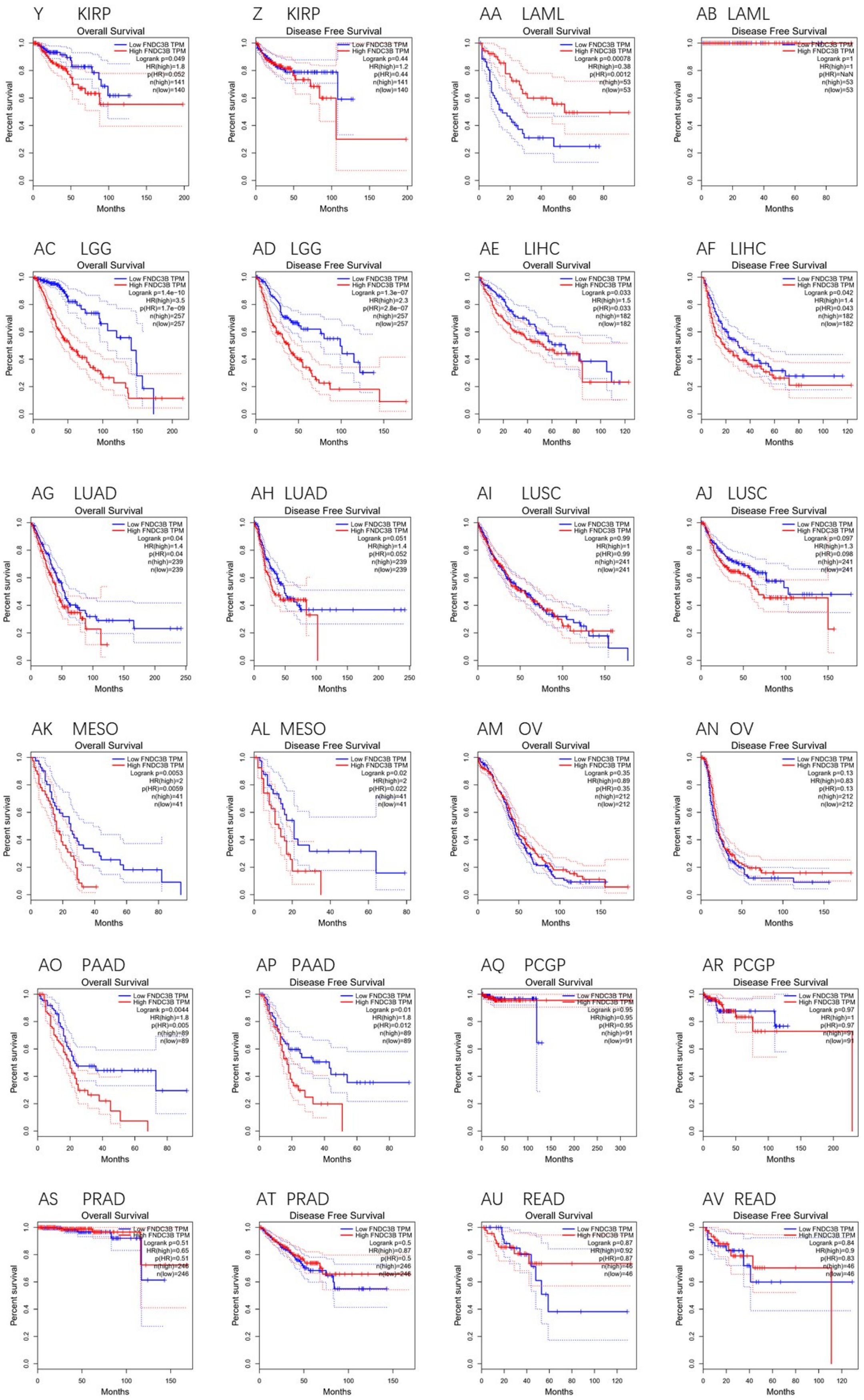
Y KIRP
Overall Survival
Low FNDC3B TPM
High FNDC3B TPM
Logrank p=0.049
HR(high)=1.8
p(HR)=0.052
n(high)=141
n(low)=140
Z KIRP
Disease Free Survival
Logrank p=0.44
HR(high)=1.2
p(HR)=0.44
n(high)=141
n(low)=140
AA LAML
Overall Survival
Logrank p=0.00078
HR(high)=0.38
p(HR)=0.0012
n(high)=53
n(low)=53
AB LAML
Disease Free Survival
Logrank p=1
HR(high)=1
p(HR)=NaN
n(high)=53
n(low)=53
AC LGG
Overall Survival
Logrank p=1.4e−10
HR(high)=3.5
p(HR)=1.7e−09
n(high)=257
n(low)=257
AD LGG
Disease Free Survival
Logrank p=1.3e−07
HR(high)=2.3
p(HR)=2.8e−07
n(high)=257
n(low)=257
AE LIHC
Overall Survival
Logrank p=0.033
HR(high)=1.5
p(HR)=0.033
n(high)=182
n(low)=182
AF LIHC
Disease Free Survival
Logrank p=0.042
HR(high)=1.4
p(HR)=0.043
n(high)=182
n(low)=182
AG LUAD
Overall Survival
Logrank p=0.04
HR(high)=1.4
p(HR)=0.04
n(high)=239
n(low)=239
AH LUAD
Disease Free Survival
Logrank p=0.051
HR(high)=1.4
p(HR)=0.052
n(high)=239
n(low)=239
AI LUSC
Overall Survival
Logrank p=0.99
HR(high)=1
p(HR)=0.99
n(high)=241
n(low)=241
AJ LUSC
Disease Free Survival
Logrank p=0.097
HR(high)=1.3
p(HR)=0.098
n(high)=241
n(low)=241
AK MESO
Overall Survival
Logrank p=0.0053
HR(high)=2
p(HR)=0.0059
n(high)=41
n(low)=41
AL MESO
Disease Free Survival
Logrank p=0.02
HR(high)=2
p(HR)=0.022
n(high)=41
n(low)=41
AM OV
Overall Survival
Logrank p=0.35
HR(high)=0.89
p(HR)=0.35
n(high)=212
n(low)=212
AN OV
Disease Free Survival
Logrank p=0.13
HR(high)=0.83
p(HR)=0.13
n(high)=212
n(low)=212
AO PAAD
Overall Survival
Logrank p=0.0044
HR(high)=1.8
p(HR)=0.005
n(high)=89
n(low)=89
AP PAAD
Disease Free Survival
Logrank p=0.01
HR(high)=1.8
p(HR)=0.012
n(high)=89
n(low)=89
AQ PCGP
Overall Survival
Logrank p=0.95
HR(high)=0.95
p(HR)=0.95
n(high)=91
n(low)=91
AR PCGP
Disease Free Survival
Logrank p=0.97
HR(high)=1
p(HR)=0.97
n(high)=91
n(low)=91
AS PRAD
Overall Survival
Logrank p=0.51
HR(high)=0.65
p(HR)=0.51
n(high)=246
n(low)=246
AT PRAD
Disease Free Survival
Logrank p=0.5
HR(high)=0.87
p(HR)=0.5
n(high)=246
n(low)=246
AU READ
Overall Survival
Logrank p=0.87
HR(high)=0.92
p(HR)=0.87
n(high)=46
n(low)=46
AV READ
Disease Free Survival
Logrank p=0.84
HR(high)=0.9
p(HR)=0.83
n(high)=46
n(low)=46
Percent survival
Months

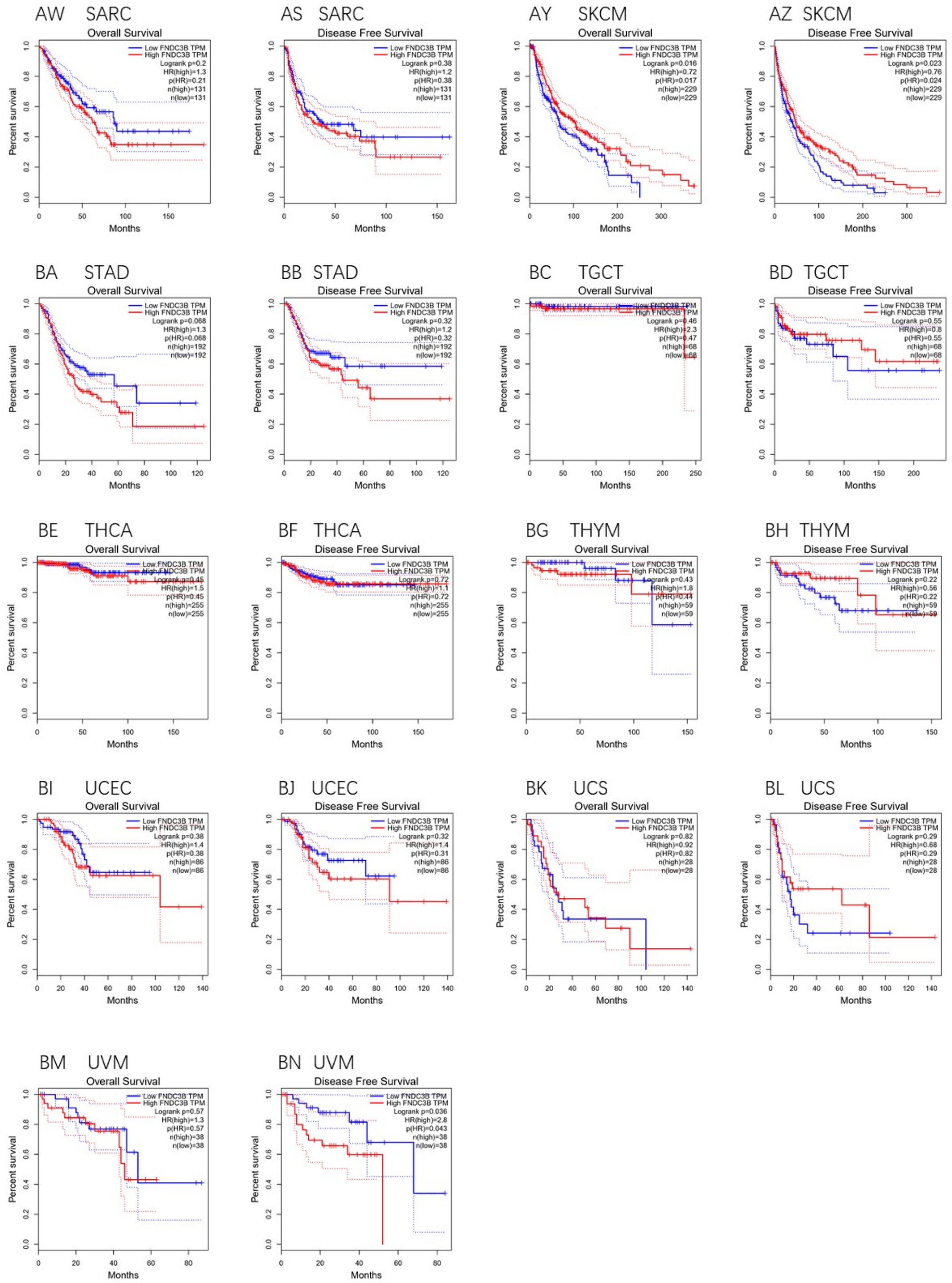
AW SARC
Overall Survival
AS SARC
Disease Free Survival
AY SKCM
Overall Survival
AZ SKCM
Disease Free Survival
BA STAD
Overall Survival
BB STAD
Disease Free Survival
BC TGCT
Overall Survival
BD TGCT
Disease Free Survival
BE THCA
Overall Survival
BF THCA
Disease Free Survival
BG THYM
Overall Survival
BH THYM
Disease Free Survival
BI UCEC
Overall Survival
BJ UCEC
Disease Free Survival
BK UCS
Overall Survival
BL UCS
Disease Free Survival
BM UVM
Overall Survival
BN UVM
Disease Free Survival
Percent survival
Months

Supplementary Figure 4. Kaplan-Meier analysis of overall survival (OS) based on high vs low expression of FNDC3B in pan-glioma patients in the CGGA data sets. (A) mRNAseq_325 data set; (B) mRNAseq_693 data set; (C) mRNA_array_301 data set. The red curve represents patients with high expression of FNDC3B, and the blue curve represents patients with low expression of FNDC3B.

(A)

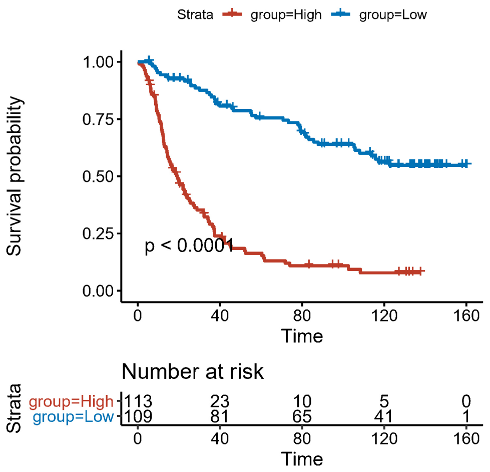

(B)

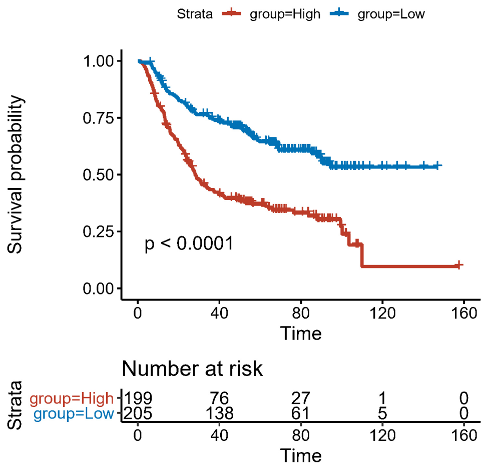

(C)

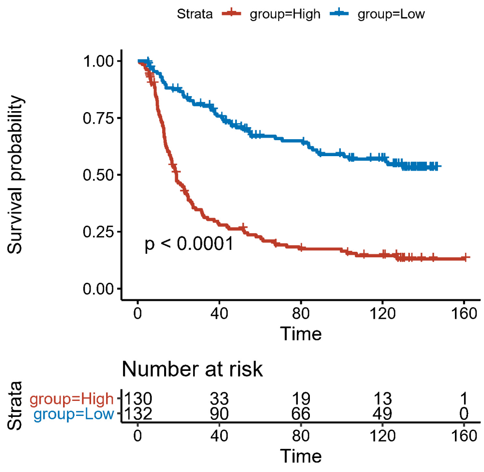

Supplementary Figure 5. Gene ontology (GO) functional annotation and hub gene analysis for the turquoise module. The top GO terms in the enrichment analysis.

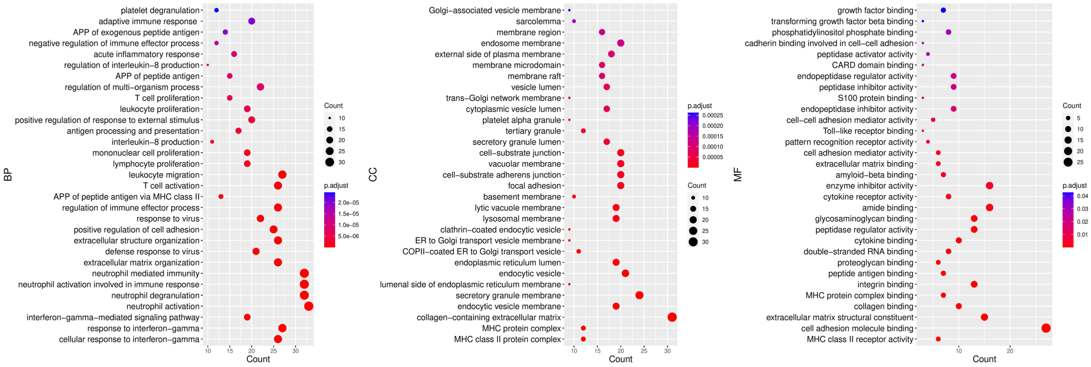

Supplementary Figure 6. 210 genes remained in the PPI network construction for the turquoise module. A total of 210 nodes and 1,061 edges were mapped for the turquoise module genes in the PPI network.

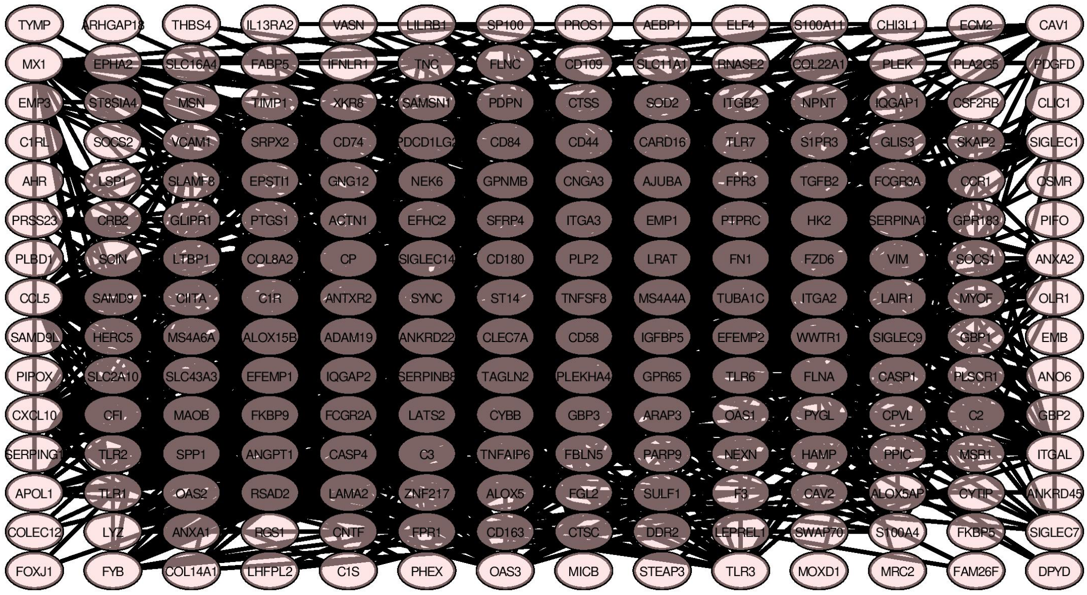

Supplementary Figure 7. High expression of the hub genes predicts shorter overall survival (OS) of low-grade glioma (LGG) patients. Kaplan-Meier curves showed that LGG patients with high expression of TLR2 (A), TLR7 (B), PTPRC (C), CCR1 (D), CCL5 (E), TLR1 (F), FN1 (G), VCAM1 (H), CXCL10 (I), and TLR6 (J) had reduced OS.

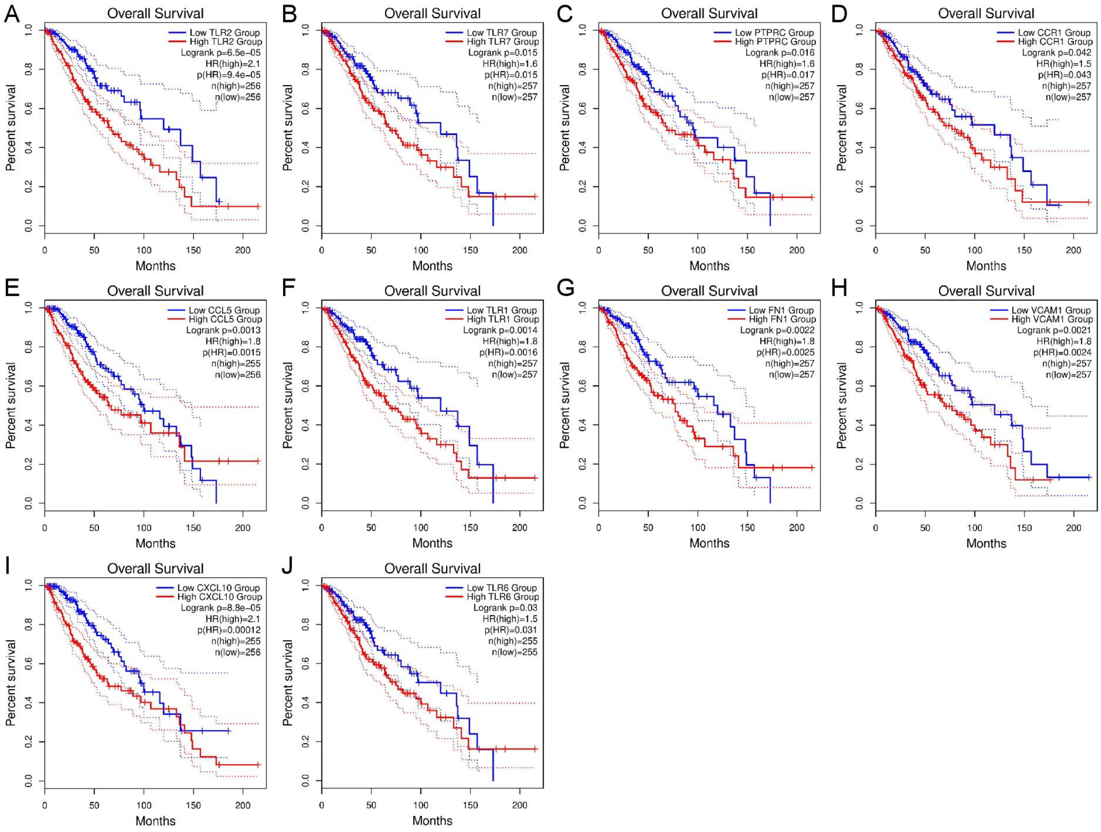

Supplement: Supplementary file 1 [file DataSheet_1.pdf]
